# Supplementary material for: The histone H3 lysine 36 demethylase KDM2A/FBXL11 controls Polycomb-mediated gene repression and germ cell development in male mice
Source: Nat Commun. 2025 Jul 23;16:6803. doi: 10.1038/s41467-025-61733-x (PMC12287389; doi:10.1038/s41467-025-61733-x)
Supplement: Supplementary file 3 — Reporting Summary [file 41467_2025_61733_MOESM3_ESM.pdf]

nature portfolio

Corresponding author(s): Antoine HFM Peters

Last updated by author(s): Jun 10, 2025

Reporting Summary

Nature Portfolio wishes to improve the reproducibility of the work that we publish. This form provides structure for consistency and transparency in reporting. For further information on Nature Portfolio policies, see our Editorial Policies and the Editorial Policy Checklist.

Statistics

For all statistical analyses, confirm that the following items are present in the figure legend, table legend, main text, or Methods section.

n/a	Confirmed	
☐	☒	The exact sample size (*n*) for each experimental group/condition, given as a discrete number and unit of measurement
☐	☒	A statement on whether measurements were taken from distinct samples or whether the same sample was measured repeatedly
☐	☒	The statistical test(s) used AND whether they are one- or two-sided<br>*Only common tests should be described solely by name; describe more complex techniques in the Methods section.*
☐	☒	A description of all covariates tested
☐	☒	A description of any assumptions or corrections, such as tests of normality and adjustment for multiple comparisons
☐	☒	A full description of the statistical parameters including central tendency (e.g. means) or other basic estimates (e.g. regression coefficient) AND variation (e.g. standard deviation) or associated estimates of uncertainty (e.g. confidence intervals)
☐	☒	For null hypothesis testing, the test statistic (e.g. *F*, *t*, *r*) with confidence intervals, effect sizes, degrees of freedom and *P* value noted<br>*Give P values as exact values whenever suitable.*
☒	☐	For Bayesian analysis, information on the choice of priors and Markov chain Monte Carlo settings
☒	☐	For hierarchical and complex designs, identification of the appropriate level for tests and full reporting of outcomes
☐	☒	Estimates of effect sizes (e.g. Cohen's *d*, Pearson's *r*), indicating how they were calculated

Our web collection on statistics for biologists contains articles on many of the points above.

Software and code

Policy information about availability of computer code

Data collection	Genomic data were collected with HiSeq 2500 (Illumina) and NextSeq 500 (Illumina) platforms.<br>Imaging data were collected with Zeiss LSM 700 confocal microscope equipped with 40x and 63×Plan-Apochromat objectives and Axioscan Z1 (Zeiss) microscopes.<br>Flow cytometry data were collected with BD FACSAria III (Becton Dickinson) cell sorter.
Data analysis	TrimGalore (version 0.6.2), STAR (version 2.5.0a), SAMtools (version 1.10), nudup.py (version 2.2), Rstudio (R version 4.2.1),<br>limma (version 3.52.2), QuasR (version 1.36.0), PCAtools (version 2.8.0), stats (version 4.2.1) , topGO (version 2.48.0), edgeR (version 3.42.4),ggplot2, ComplexHeatmaps, ImageJ (Fiji version 1.53f51), FACS BD Diva (version 9), FlowJo (version 10),GraphPad Prism Software Version 7.0, Aperio ePathology Solutions; Leica Biosystems, Loupe Browser (v8.1.2, 10x Genomics)

For manuscripts utilizing custom algorithms or software that are central to the research but not yet described in published literature, software must be made available to editors and reviewers. We strongly encourage code deposition in a community repository (e.g. GitHub). See the Nature Portfolio guidelines for submitting code & software for further information.

Data

Policy information about availability of data

All manuscripts must include a data availability statement. This statement should provide the following information, where applicable:

- Accession codes, unique identifiers, or web links for publicly available datasets
- A description of any restrictions on data availability
- For clinical datasets or third party data, please ensure that the statement adheres to our policy

The datasets produced in this study are available in the following database: Gene Expression Omnibus GSE214682 (https://www.ncbi.nlm.nih.gov/geo/query/acc.cgi?acc=GSE214682)
Imaging and flow cytometry data are available from the corresponding authors on reasonable request.

Research involving human participants, their data, or biological material

Policy information about studies with human participants or human data. See also policy information about sex, gender (identity/presentation), and sexual orientation and race, ethnicity and racism.

Reporting on sex and gender	Not Applicable
Reporting on race, ethnicity, or other socially relevant groupings	Not Applicable
Population characteristics	Not Applicable
Recruitment	Not Applicable
Ethics oversight	Not Applicable

Note that full information on the approval of the study protocol must also be provided in the manuscript.

Field-specific reporting

Please select the one below that is the best fit for your research. If you are not sure, read the appropriate sections before making your selection.

[x] Life sciences [ ] Behavioural & social sciences [ ] Ecological, evolutionary & environmental sciences

For a reference copy of the document with all sections, see nature.com/documents/nr-reporting-summary-flat.pdf

Life sciences study design

All studies must disclose on these points even when the disclosure is negative.

Sample size	For whole body histopathology analysis and body weight measurements the initial cohort included twelve 10-week old iKO mice and twelve control (Ctrl) littermates equally distributed between genders<br>For testis histopathology and immunohistochemistry experiments the cohorts included 3 iKO mice and 3 Ctrl littermates<br>For bulk RNAseq, RRBS and anti-H3K27me3 ChiPseq, 4 replicates for each biological condition were performed.<br>For anti-H3K36me2 and anti-H3K4me3 ChIPseq, 2 biological replicates for each biological condition were performed.<br>The sample sizes were determined based on our previous experimental experience in which such numbers could give cost efficient and reliable result for downstream analysis.
Data exclusions	No data were excluded.
Replication	*Describe the measures taken to verify the reproducibility of the experimental findings. If all attempts at replication were successful, confirm this OR if there are any findings that were not replicated or cannot be reproduced, note this and describe why.*
Randomization	Randomization was not relevant for this study.
Blinding	Blinding was not relevant for the experimental set up. Blinding was not relevant to this study because it involved automated data collection [NGS sequencing] and automated retrospective data analysis [counting of reads was performed by software in R] that do not require subjective assessment. The investigators were not blinded to allocation during histology experiments and outcome assessment.

Reporting for specific materials, systems and methods

We require information from authors about some types of materials, experimental systems and methods used in many studies. Here, indicate whether each material, system or method listed is relevant to your study. If you are not sure if a list item applies to your research, read the appropriate section before selecting a response.

Materials & experimental systems

n/a	Involved in the study
☐	☒ Antibodies
☒	☐ Eukaryotic cell lines
☒	☐ Palaeontology and archaeology
☐	☒ Animals and other organisms
☒	☐ Clinical data
☒	☐ Dual use research of concern
☒	☐ Plants

Methods

n/a	Involved in the study
☐	☒ ChIP-seq
☐	☒ Flow cytometry
☒	☐ MRI-based neuroimaging

Antibodies

Antibodies used

For chromogenic immunohistochemistry :
anti-PLZF (Active Motif, #39987 at 20 μg/ml)
anti-c-KIT (Cell Signaling, #CST3074 at 0.10 μg/ml)
anti-γH2A.X (Cell Signaling, #CST9718 at 0.145 μg/ml)
anti-TRA98 (Abcam, ab82527 at 0.5 μg/ml)

For immunofluorescence :
anti-PLZF (Active Motif, #39987, 1:50)
anti-CD117/c-kit (R&D systems, AF1356-SP, 1:1000)
anti-KDM2A (Abcam, ab191387, 1:1000)
Lectin PNA Alexa Fluor™ 647 Conjugate (Thermo Fisher, L32460, 1:1000)
anti-SOX9 (Millipore, #AB5535, 1:300)

For immunofluorescence of meiotic chromosoem spreads:
anti-H3K4me3 (Millipore, 17-614, 1:200)
anti-H3K9me2 (Abcam, ab1220, 1:300)
anti-H3K9me3 (Invitrogen, 49-1008, 1:200)
anti-SCP3 (Abcam, ab15093, 1:1000)
guinea pig anti-HORMAD1 (homemade Wojtasz, et al. (2009) PLoS genetics, 1:100)
anti-γH2A.X (Millipore, 05636, 1:5000)
anti-ATR (Santa Cruz, sc-1887, 1:200)

For FACS:
anti-CD117/c-kit PE (eBioscience 12-1171-83, 1:200)
anti-CD324/E-Cadherin eFluor 660 (eBioscience 50-3249-82, 1:200)
anti-CD49f/Integrin alpha 6 PE-Cyanine7 (eBioscience 25-0459-82, 1:200)

For ChIPseq:
anti-H3K4me3 (Millipore, 17-614, 0.5 μg/sample)
anti-H3K36me2 (Upstate, 07-369, 1 μg/sample)
anti-H3K27me3 (Cell Signaling, 9733, 0.75 μg/sample)

Validation

anti-PLZF (Active Motif, #39987)
Applications: IP, ICC/IF, WB

anti-c-KIT (Cell Signaling, #CST3074)
Applications: IP, IF, WB

anti-γH2A.X (Cell Signaling, #CST9718)
Applications: F, IHC, IF, WB

anti-TRA98 (Abcam, ab82527)
Applications: IHC, WB

anti-CD117/c-kit (R&D systems, AF1356-SP)
Applications: WB, F, IHC, CyTOF-ready

anti-KDM2A (Abcam, ab191387)
IP, WB, ICC/IF, IHC-P

anti-SOX9 (Millipore, #AB5535)
IHC, WB, ChIP, ChIP-seq, ICC, IF

anti-H3K4me3 (Millipore, 17-614)
Applications: WB, ChIP, ChIP-seq

anti-H3K9me2 (Abcam, ab1220)
Applications: ICC/IF, ChIP, ELISA, WB, IHC-P

anti-H3K9me3 (Invitrogen, 49-1008)
Applications:WB, ICC/IF, ELISA, ChIP, Peptide Array, DB

anti-SCP3 (Abcam, ab15093)
Applications: IHC, ICC/IF

guinea pig anti-HORMAD1 (homemade Wojtasz, et al. (2009) PLoS genetics, 1:100)
Applications: IF

anti-γH2A.X (Millipore, 05636)
Applications: ICC, IF, WB, ChIP, IHC

anti-CD117/c-kit PE (eBioscience 12-1171-83) :
Applications: IHC, F

anti-CD324/E-Cadherin eFluor 660 (eBioscience 50-3249-82):
Applications: WB, IHC, ICC/IF, F, Neutralization

anti-CD49f/Integrin alpha 6 PE-Cyanine7 (eBioscience 25-0459-82):
Applications: F

anti-H3K36me2 (Upstate, 07-369)
Applications: WB, DB, ChIP

anti-H3K27me3 (Cell Signaling, 9733)
Applications: WB, IHC, IF, F, ChIP, C&R, C&T
anti-H3K36me3 (Cell Signaling 4909)
Applications: WB, IHC, IF, F, ChIP, C&R

anti-H3K36me2 (CosmoBio MABI0332)
Applications: WB, ICC, ChIP

Legend:
IP:Immunoprecipitation
WB:Western Blotting
IHC: Immunohistochemistry
ICC: Immunocytochemistry
IF: Immunofluorescence
F: Flow Cytometry
ChIP: Chromatin Immunoprecipitation
DB: Dot Blot
C&R: CUT & RUN
C&T: CUT & Tag

Animals and other research organisms

Policy information about studies involving animals; ARRIVE guidelines recommended for reporting animal research, and Sex and Gender in Research

Laboratory animals

Mice bearing Kdm2a flox alleles were generated during this study in C57BL/6J background.

Mice bearing the CreERTM expressing transgene under the control of the CMV-IE enhancer/chicken β-actin/rabbit β-globin hybrid promoter were obtained from The Jackson Laboratory; JAX stock number: 004682

Mice bearing the CreERTM transgene expressed from the endogenous Actb locus

Mice bearing the Cre expressing transgene under the control of the Ngn3 promoter were obtained from The Jackson Laboratory; JAX stock number: 006333

To determine the effect of Kdm2a loss in adult mice, we administered tamoxifen on five sequential days to twelve 10-week old iKO mice and twelve control (Ctrl) littermates (genotype Kdm2a2lox/2lox) equally distributed between genders, and carefully monitored

them for 65 days. For the time course experiment we conducted a 15-day time course experiment, collecting testes every 3 days following the start of tamoxifen administration, using 11 males 10-week old iKO mice and 11 control (Ctrl) littermates. For the genomic and most of the immunofluorescence experiments the start of tamoxifen administration was performed using 6 males 16-week old iKO mice and 6 control (Ctrl) littermates for 10 days.

Wild animals	No wild animals used in this study.
Reporting on sex	Male mice were primarily used in this study. Female mice were used in the initial cohort.
Field-collected samples	No field collected samples used in this study.
Ethics oversight	All animal procedures employed in this study were approved by the Novartis Institutes for BioMedical Research Institutional Animal Care and Use Committee (Protocol Numbers: 11 DP 037 and 14 DMP 049), conformed to the Swiss Animal Protection Ordinance (protocol number: 2612) and are compliant with the FMI institutional guidelines.

Note that full information on the approval of the study protocol must also be provided in the manuscript.

Plants

Seed stocks	not applicable
Novel plant genotypes	not applicable
Authentication	not applicable

ChIP-seq

Data deposition

☒ Confirm that both raw and final processed data have been deposited in a public database such as GEO.

☐ Confirm that you have deposited or provided access to graph files (e.g. BED files) for the called peaks.

Data access links
May remain private before publication.

We submitted genomic data sets to GEO where they are available using the following token: uxyvuqoirxwnhkh. The token provides anonymous, read-only access to GSE214682 and associated accessions (https://www.ncbi.nlm.nih.gov/geo/query/acc.cgi?acc=GSE214682) .

Files in database submission

anti-H3K4me3 ChIP-seq:
GSM6614151 H3K4me3_SgD_KDM2A_CTR_r1
GSM6614152 H3K4me3_SgD_KDM2A_CTR_r2
GSM6614153 H3K4me3_SgD_KDM2A_iKO_r1
GSM6614154 H3K4me3_SgD_KDM2A_iKO_r2

anti-H3K36me2 ChIP-seq:
GSM6614155 H3K36me2_SgD_KDM2A_CTR_r1
GSM6614156 H3K36me2_SgD_KDM2A_CTR_r2
GSM6614157 H3K36me2_SgD_KDM2A_iKO_r1
GSM6614158 H3K36me2_SgD_KDM2A_iKO_r2

anti-H3K27me3 ChIP-seq:
GSM6614159 H3K27me3_SgD_KDM2A_CTR_r1
GSM6614160 H3K27me3_SgD_KDM2A_CTR_r2
GSM6614161 H3K27me3_SgD_KDM2A_CTR_r3
GSM6614162 H3K27me3_SgD_KDM2A_CTR_r4
GSM6614163 H3K27me3_SgD_KDM2A_iKO_r1
GSM6614164 H3K27me3_SgD_KDM2A_iKO_r2
GSM6614165 H3K27me3_SgD_KDM2A_iKO_r3
GSM6614166 H3K27me3_SgD_DM2A_iKO_r4
GSM8800242 K27m3_SgU_WT_r1
GSM8800243 K27m3_SgU_WT_r2
GSM8800244 K27m3_SgD_WT_r1
GSM8800245 K27m3_SgD_WT_r2
GSM8800246 K27m3_ScLZ_WT_r1

GSM8800247 K27m3_ScLZ_WT_r2
GSM8800248 K27m3_ScPD_WT_r1
GSM8800249 K27m3_ScPD_WT_r2

RNA-seq:
GSM6614167 RNA_ScLZ_KDM2A_CTR_r1
GSM6614168 RNA_ScLZ_KDM2A_CTR_r2
GSM6614169 RNA_ScLZ_KDM2A_CTR_r3
GSM6614170 RNA_ScLZ_KDM2A_CTR_r4
GSM6614171 RNA_ScLZ_KDM2A_iKO_r1
GSM6614172 RNA_ScLZ_KDM2A_iKO_r2
GSM6614173 RNA_ScLZ_KDM2A_iKO_r3
GSM6614174 RNA_ScLZ_KDM2A_iKO_r4
GSM6614175 RNA_SgU_KDM2A_CTR_r1
GSM6614176 RNA_SgU_KDM2A_CTR_r2
GSM6614177 RNA_SgU_KDM2A_CTR_r3
GSM6614178 RNA_SgU_KDM2A_CTR_r4
GSM6614179 RNA_SgU_KDM2A_iKO_r1
GSM6614180 RNA_SgU_KDM2A_iKO_r2
GSM6614181 RNA_SgU_KDM2A_iKO_r3
GSM6614182 RNA_SgU_KDM2A_iKO_r4
GSM6614183 RNA_SgD_KDM2A_CTR_r1
GSM6614184 RNA_SgD_KDM2A_CTR_r2
GSM6614185 RNA_SgD_KDM2A_CTR_r3
GSM6614186 RNA_SgD_KDM2A_CTR_r4
GSM6614187 RNA_SgD_KDM2A_iKO_r1
GSM6614188 RNA_SgD_KDM2A_iKO_r2
GSM6614189 RNA_SgD_KDM2A_iKO_r3
GSM6614190 RNA_SgD_KDM2A_iKO_r4

Reduced Representation Bisulfite Sequencing (RRBS):
GSM6614191 RRBS_SgD_KDM2A_CTR_r1
GSM6614192 RRBS_SgD_KDM2A_CTR_r2
GSM6614193 RRBS_SgD_KDM2A_CTR_r3
GSM6614194 RRBS_SgD_KDM2A_CTR_r4
GSM6614195 RRBS_SgD_KDM2A_iKO_r1
GSM6614196 RRBS_SgD_KDM2A_iKO_r2
GSM6614197 RRBS_SgD_KDM2A_iKO_r3
GSM6614198 RRBS_SgD_KDM2A_iKO_r4

Genome browser session
(e.g. UCSC)

https://genome.ucsc.edu/s/fanogrig/Bocker_Collection

Methodology

Replicates

For bulk RNAseq, RRBS and anti-H3K27me3 ChiPseq 4 replicates for each biological condition were performed.
For anti-H3K36me2 and anti-H3K4me3 ChIPseq 2 biological replicates for each biological condition were performed.

Sequencing depth

For RRBS experiment:
umber of sequenced reads per sample min: 23.685.957, max: 26.287.209, median: 25.650.112.
mapping rate to mm10 genome min: 71.12%, max: 75.73%, median: 74.25%.

For the ChIP experiments:
number of sequenced reads per sample min: 2.066.368, max: 55.856.211, median: 31.188.428
mapping rate to mm10 genome min: 83.75%, max: 93.31%, median: 90.25%.

For RNA-seq experiment:
number of sequenced reads per sample min: 22.530.688, max: 42.003.250, median: 32.194.965
mapping rate to mm10 genome min: 61.59%, max: 80.22%, median: 75.15%.

Antibodies

anti-H3K4me3 (Millipore, 17-614, 0.5 μg/sample)
anti-H3K36me2 (Upstate, 07-369, 1 μg/sample)
anti-H3K27me3 (Cell Signaling, 9733, 0.75 μg/sample)

Peak calling parameters

ChIP-seq reads were first processed using TrimGalore (version 0.6.2) to trim adaptor and low-quality reads with settings (--stringency 3). Trimmed reads were then aligned to the mouse genome build mm10 using STAR (version 2.5.0a) with settings (--alignIntronMin 1 --alignIntronMax 1 --alignEndsType EndToEnd --alignMatesGapMax 1000 --outFilterMatchNminOverLread 0.85). No peak calling was

	performed.
Data quality	*Describe the methods used to ensure data quality in full detail, including how many peaks are at FDR 5% and above 5-fold enrichment.*
Software	TrimGalore (version 0.6.2), STAR (version 2.5.0a), Bismark (version 0.23.3), SAMtools (version 1.10), nudup.py (version 2.2), macs3 (version 3.0.0b1), Rstudio (R version 4.2.1), limma (version 3.52.2), QuasR (version 1.36.0), SCORPIUS (v1.0.8), PCAtools (version 2.8.0), stats (version 4.2.1) , topGO (version 2.48.0), monaLisa (version 1.10.1), edgeR (version 3.42.4)

Flow Cytometry

Plots

Confirm that:

☒ The axis labels state the marker and fluorochrome used (e.g. CD4-FITC).

☒ The axis scales are clearly visible. Include numbers along axes only for bottom left plot of group (a 'group' is an analysis of identical markers).

☒ All plots are contour plots with outliers or pseudocolor plots.

☒ A numerical value for number of cells or percentage (with statistics) is provided.

Methodology

Sample preparation	Testicular cell suspension was prepared by incubation of seminiferous tubules in 200 U/ml Collagenase type I (Worthington Biochemical LS004196), 5 μg/ml DNAse I (Roche 10104159001), and 0.05%Trypsin (Gibco 25200056) in GBSS (Sigma G9779) as described in "Gaysinskaya, V., Soh, I.Y., van der Heijden, G.W. and Bortvin, A. (2014) Optimized flow cytometry isolation of murine spermatocytes. Cytometry A, 85, 556-565.83". Cells were stained with 1/200 anti-CD117/c-kit PE (eBioscience 12-1171-83), anti-CD324/E-Cadherin eFluor 660 (eBioscience 50-3249-82) and anti-CD49f/Integrin alpha 6 PE-Cyanine7 (eBioscience 25-0459-82) for 1 hour at 32oC with constant shaking and protected from light. After washing cells were incubated with 20 μg/ml with Hoechst 33342 (Thermo Fischer Scientific H3570) for 1 hour at 32oC with constant shaking and protected from light and then with 30nM DRAQ7 (Biostatus DR71000) for 5 minutes on bench. The stained cells were strained through a 40μm nylon filter into 5 ml polypropylene tubes.
Instrument	BD FACSAria III cell sorter (Becton Dickinson)
Software	BD FACSDiva (version 9), FlowJo (version 10)
Cell population abundance	Undifferentiated spermatogonia are 0.2% to 0.4% of the testicular suspension.<br>Differentiated spermatogonia are 0.2% to 1% of the testicular suspension.<br>Leptotene/Zygotene spermatocytes are 0.8% to 1.6% of the testicular suspension.<br>Purity of the spermatogonia and spermatocyte sorted populations was assessed by RNAseq expression of specific marker genes and immunocytochemistry.
Gating strategy	The stained cells were strained through a 40μm nylon filter into 5 ml polypropylene tubes and were sorted on a BD FACSAria III cell sorter fitted with a 70μm nozzle (Becton Dickinson) using a 375nm laser to excite Hoechst 33342, a 561nm laser to excite PE and PE-Cy7 and a 633nm laser to excite DRAQ7 and eFluor600. Cells were first gated for FSC and SSC to exclude debris. Then the live cells were selected based on the absence of DRAQ7 signal detected using 755LP, 780/60BP. Then we gated for cells positive for Hoechst 33342 emission which was detected using a 670LP (Hoechst-Red) and 450/20 BP (Hoechst-Blue). We gated for the Hoechst-Redlow and Hoechst-Bluemid population which is enriched for 2N spermatogonia. Fluorescence for PE was detected using a 582/15BP filter, for PE-Cy7 was detected using 735LP,780/60BP filter and for eFluor660 using a 660/20BP filter. Undifferentiated spermatogonia were sorted as CD324high, CD49fhigh, CD117low and differentiated spermatogonia were sorted as CD324low, CD49flow, CD117high. We gated for the Hoechst-Redmid and Hoechst-Bluehigh population which is enriched for Leptotene and Zygotene spermatocytes

☒ Tick this box to confirm that a figure exemplifying the gating strategy is provided in the Supplementary Information.
