# Supplementary material for: The histone H3 lysine 36 demethylase KDM2A/FBXL11 controls Polycomb-mediated gene repression and germ cell development in male mice
Source: Nat Commun. 2025 Jul 23;16:6803. doi: 10.1038/s41467-025-61733-x (PMC12287389; doi:10.1038/s41467-025-61733-x)
Supplement: Supplementary file 1 — Supplementary Information [file 41467_2025_61733_MOESM1_ESM.pdf]

SUPPLEMENTARY FIGURES

The histone H3 lysine 36 demethylase KDM2A/FBXL11 controls Polycomb-mediated gene repression and germ cell development in male mice

Michael T. Bocker, Grigorios Fanourgakis, Kristie Wetzel, Pavel A. Komarov, Hélène Royo, Alexia Romer, Sunwoo Chun, Ching-Yeu Liang, Hubertus Kohler, Taiping Chen, Xiaohong Mao, Mark A. Labow, Reginald A. Valdez, Michael B. Stadler, Dirk G. de Rooij, Paola Capodieci, John Tallarico, Antoine H. F. M. Peters, Thomas B. Nicholson

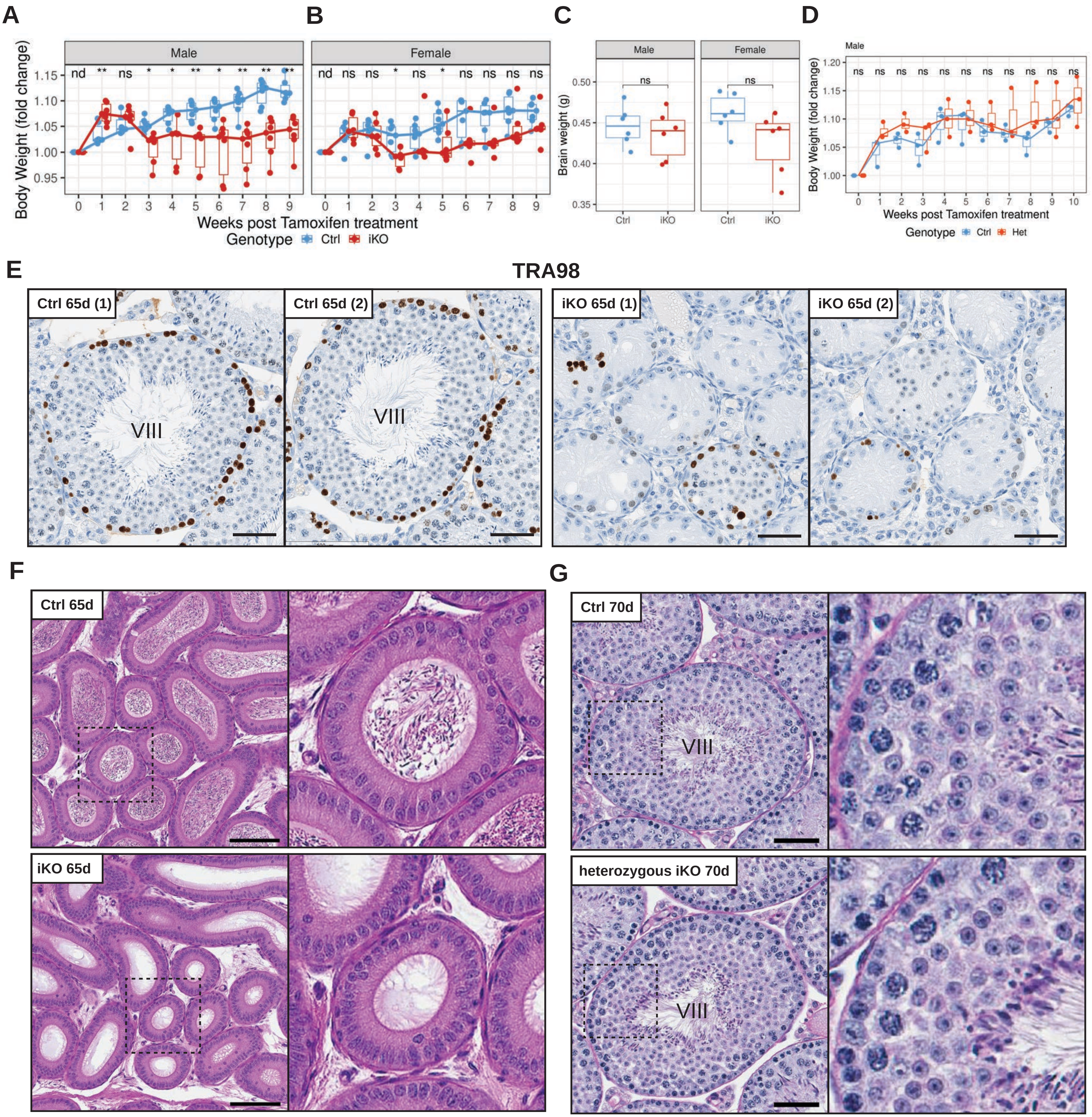
A
B
C
D
Male
Female
Body Weight (fold change)
1.15
1.10
1.05
1.00
0.95
nd ** ns * * ** * ** ** **
nd ns ns * ns * ns ns ns ns
0 1 2 3 4 5 6 7 8 9
Weeks post Tamoxifen treatment
Genotype Ctrl iKO
Brain weight (g)
0.50
0.45
0.40
0.35
ns
Ctrl iKO
Male
1.20
1.15
1.10
1.05
1.00
ns ns ns ns ns ns ns ns ns ns ns
0 1 2 3 4 5 6 7 8 9 10
Weeks post Tamoxifen treatment
Genotype Ctrl Het
E
TRA98
Ctrl 65d (1)
Ctrl 65d (2)
iKO 65d (1)
iKO 65d (2)
VIII
VIII
F
Ctrl 65d
iKO 65d
G
Ctrl 70d
heterozygous iKO 70d
VIII
VIII

Supplementary Figure 1. Characterization of *Kdm2a* iKO phenotype and heterozygous *Kdm2a* testes.

(A, B) Body weight of Ctrl (*Kdm2a*$^{2lox/2lox}$) and *Kdm2a* inducible knockout (iKO: *Kdm2a*$^{2lox/2lox}$; *Actb*$^{CreERT2/+}$) males (A) and females (B) relative to body weight at day 0 of tamoxifen treatment. n = 6 per group. Two-sample, two-sided Wilcoxon tests were performed between indicated groups (ns) P>0.05; (*) P < 0.05; (**) P < 0.01;. Center lines in box plots represent median values while lower and upper lines indicate the interquartile range (IQR; from the 25th to 75th percentile).

(C) Brain weights of Ctrl (*Kdm2a*$^{2lox/2lox}$) and *Kdm2a* inducible knockout (iKO: *Kdm2a*$^{2lox/2lox}$; *Actb*$^{CreERT2/+}$) males and females at day 65 of tamoxifen treatment. n= 6 per group. Two-sample, two-sided Wilcoxon tests were performed between indicated groups (ns) P>0.05. Center lines in box plots represent median values while lower and upper lines indicate the interquartile range (IQR; from the 25th to 75th percentile).

(D) Body weight of heterozygous Ctrl (*Kdm2a*$^{2lox/+}$) and heterozygous iKO (*Kdm2a*$^{2lox/+}$;*Actb*$^{CreERT2/+}$) males relative to body weight at day 0 of tamoxifen treatment. n = 3 per group. Two-sample, two-sided Wilcoxon tests were performed between indicated groups (ns) P>0.05. Center lines in box plots represent median values while lower and upper lines indicate the interquartile range (IQR; from the 25th to 75th percentile).

(E) Immunohistochemical staining of stage VIII seminiferous tubules from 65d testes from Ctrl and *Kdm2a* iKO males with an anti-TRA98 antibody and a hematoxylin counter stain. Scale bar = 50μm.

(F) Hematoxylin and eosin staining of Ctrl and *Kdm2a* iKO caudal epididymis at 65d. Boxed regions in left panels are magnified to the right. Scale bar = 100μm.

(G) PAS-stained sections showing stage VIII seminiferous tubules of heterozygous Ctrl (*Kdm2a*$^{2lox/+}$) and heterozygous iKO (*Kdm2a*$^{2lox/+}$;*Actb*$^{CreERT2/+}$) males at 70d post first tamoxifen treatment. Boxed regions in left panels are magnified to the right. Scale bar = 50μm.

A

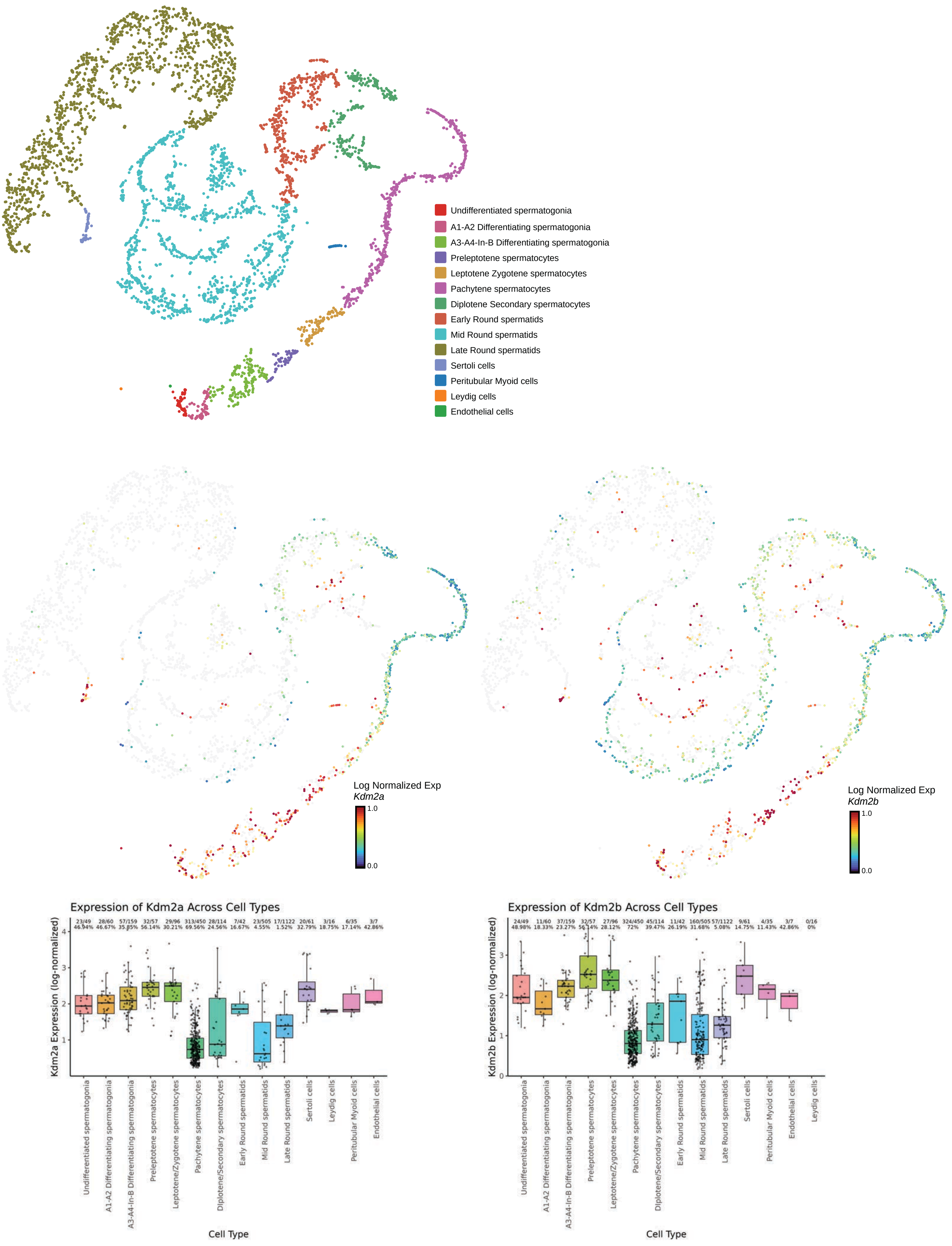

Undifferentiated spermatogonia
A1-A2 Differentiating spermatogonia
A3-A4-In-B Differentiating spermatogonia
Preleptotene spermatocytes
Leptotene Zygotene spermatocytes
Pachytene spermatocytes
Diplotene Secondary spermatocytes
Early Round spermatids
Mid Round spermatids
Late Round spermatids
Sertoli cells
Peritubular Myoid cells
Leydig cells
Endothelial cells
Log Normalized Exp
Kdm2a
1.0
0.0
Log Normalized Exp
Kdm2b
1.0
0.0
Expression of Kdm2a Across Cell Types
23/49 46.94%
28/60 46.67%
57/159 35.85%
32/57 56.14%
29/96 30.21%
313/450 69.56%
28/114 24.56%
7/42 16.67%
23/505 4.55%
17/1122 1.52%
20/61 32.79%
3/16 18.75%
6/35 17.14%
3/7 42.86%
Kdm2a Expression (log-normalized)
4
3
2
1
Undifferentiated spermatogonia
A1-A2 Differentiating spermatogonia
A3-A4-In-B Differentiating spermatogonia
Preleptotene spermatocytes
Leptotene/Zygotene spermatocytes
Pachytene spermatocytes
Diplotene/Secondary spermatocytes
Early Round spermatids
Mid Round spermatids
Late Round spermatids
Sertoli cells
Leydig cells
Peritubular Myoid cells
Endothelial cells
Cell Type
Expression of Kdm2b Across Cell Types
24/49 48.98%
11/60 18.33%
37/159 23.27%
32/57 56.14%
27/96 28.12%
324/450 72%
45/114 39.47%
11/42 26.19%
160/505 31.68%
57/1122 5.08%
9/61 14.75%
4/35 11.43%
3/7 42.86%
0/16 0%
Kdm2b Expression (log-normalized)
3
2
1
0
Undifferentiated spermatogonia
A1-A2 Differentiating spermatogonia
A3-A4-In-B Differentiating spermatogonia
Preleptotene spermatocytes
Leptotene/Zygotene spermatocytes
Pachytene spermatocytes
Diplotene/Secondary spermatocytes
Early Round spermatids
Mid Round spermatids
Late Round spermatids
Sertoli cells
Peritubular Myoid cells
Endothelial cells
Leydig cells
Cell Type

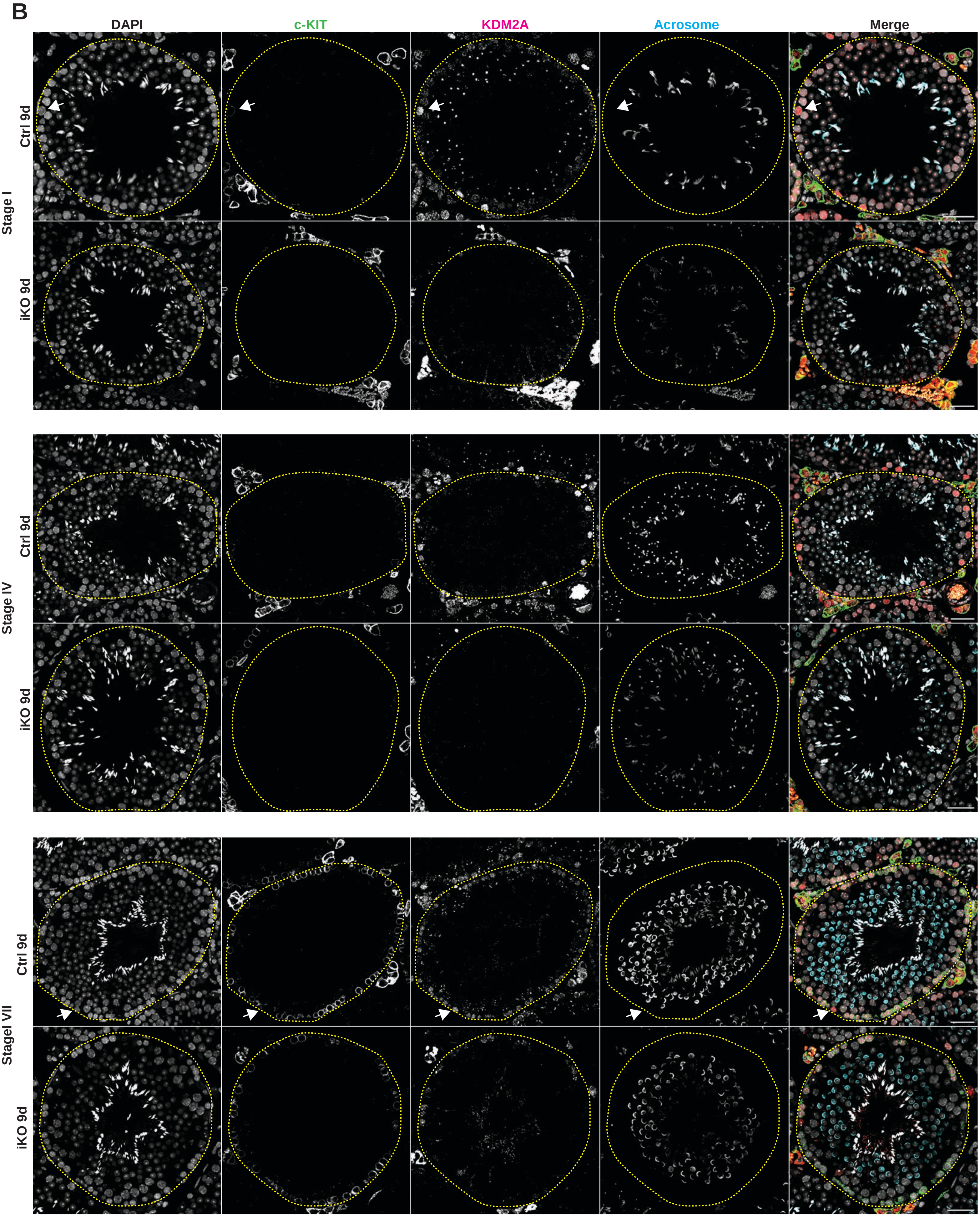
B
DAPI
c-KIT
KDM2A
Acrosome
Merge
Stage I
Ctrl 9d
iKO 9d
Stage IV
Ctrl 9d
iKO 9d
StageI VII
Ctrl 9d
iKO 9d

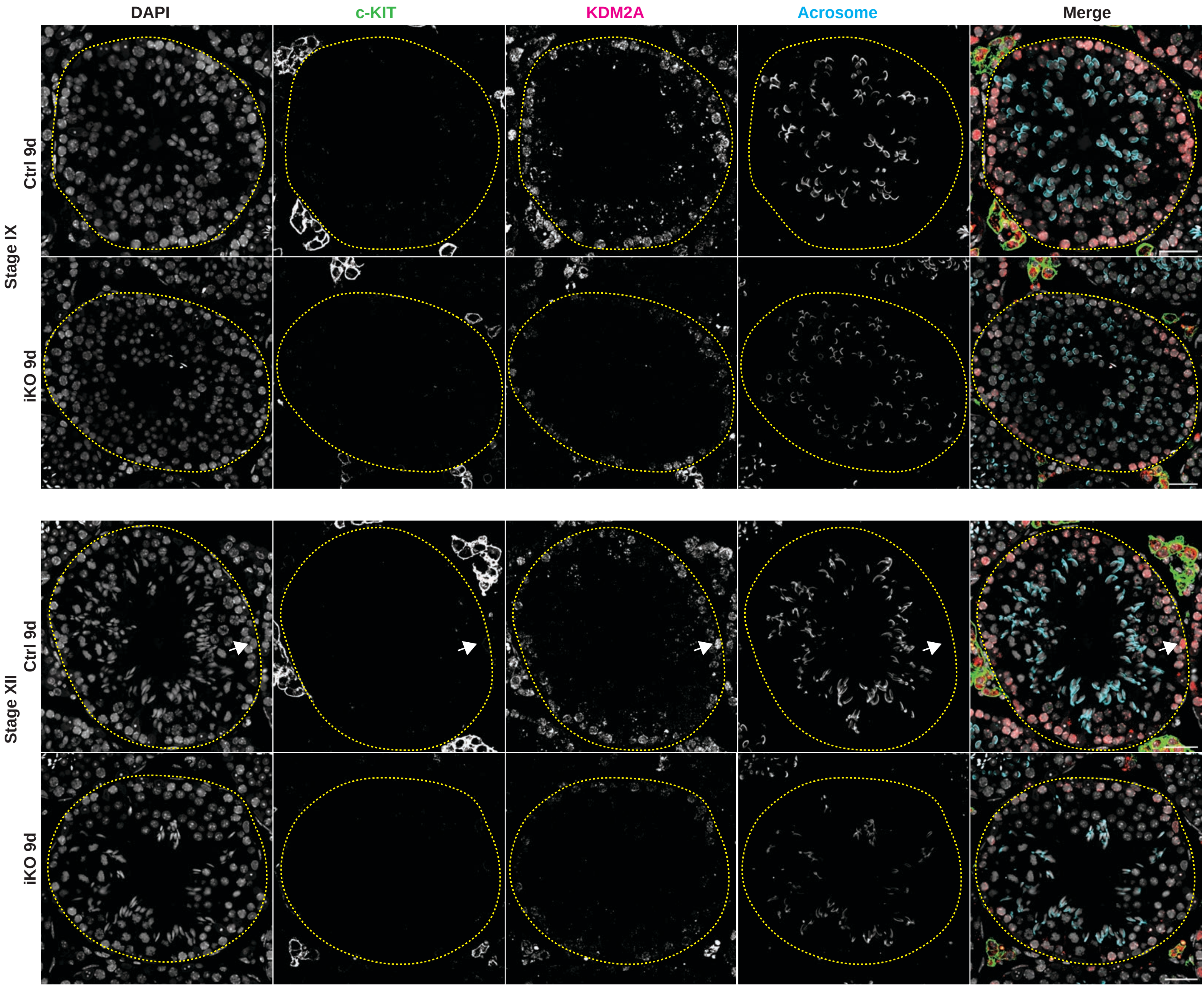

DAPI
c-KIT
KDM2A
Acrosome
Merge
Stage IX
Ctrl 9d
iKO 9d
Stage XII
Ctrl 9d
iKO 9d

Supplementary Figure 2. Stage dependent presence of KDM2A in Ctrl and *Kdm2a* iKO

A) t-SNE plots showing the different cell clusters defined by Herman *et al.* (73) and the log2 normalized expression values of *Kdm2a and Kdm2b* (top). Boxplots showing the log-normalized expression of *Kdm2a and Kdm2b* in different spermatogenic and somatic cell populations as defined by Hermann *et al.* (73). The numbers and percentages of cells assigned to a cell type expressing *Kdm2a and Kdm2b* are indicated above the boxplots (bottom).

B) Immunofluorescence staining of testicular sections from Ctrl (*Kdm2a*$^{2lox/2lox}$) (n=1) and *Kdm2a* inducible knockout (iKO: *Kdm2a*$^{2lox/2lox}$; *CAGG-Cre-ER*^{TM}) (n=1) at 9d post first tamoxifen treatment with an anti-c-KIT (green), anti-KDM2A (red) antibodies. The acrosome and nucleus were counter stained with Lectin PNA (blue) and DAPI (white) respectively. Representative seminiferous tubules of various stages are encircled with yellow dashed lines. White arrows point to c-KIT-low spermatogonia. Scale bar = 50μm.

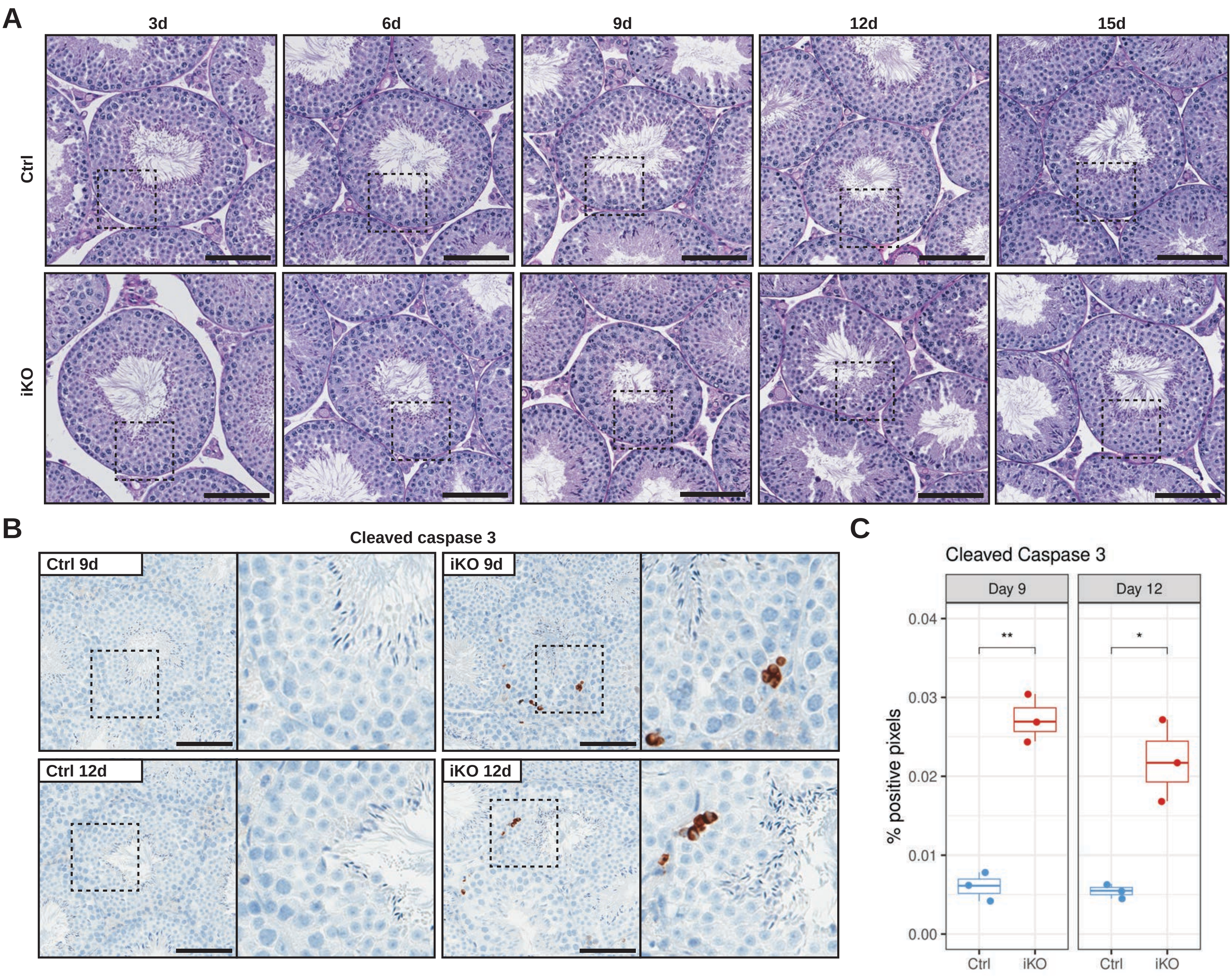

A
3d
6d
9d
12d
15d
Ctrl
iKO
B
Cleaved caspase 3
Ctrl 9d
iKO 9d
Ctrl 12d
iKO 12d
C
Cleaved Caspase 3
Day 9
Day 12
% positive pixels
0.04
0.03
0.02
0.01
0.00
**
*
Ctrl
iKO
Ctrl
iKO

Supplementary Figure 3. Characterization of apoptotic cells in *Kdm2a* iKO.

(A) PAS-stained sections showing stage VIII seminiferous tubules of control (*Kdm2a*$^{2lox/2lox}$) and *Kdm2a* inducible knockout (iKO: *Kdm2a*$^{2lox/2lox}$; *Actb*$^{CreERT2/+}$) males at 3d, 6d, 9d, 12d, and 15d post first Tamoxifen treatment. n = 1 for 3d and 6d; n = 3 for subsequent time points. Boxed regions are shown in Fig. 2A. Scale bar = 100μm.

(B) Immunohistochemical staining of testes from Ctrl and *Kdm2a* iKO males at 9d and 12d with an anti-cleaved Caspase 3 antibody (brown) and with hematoxylin counter stain. n = 3 per group. Scale bar = 50μm.

(C) Quantitation of cleaved Caspase 3 staining of seminiferous tubules from testes of Ctrl (n=3) and iKO (n=3) mice at 9d and 12d. Percent of positive pixels for the entire cross section of testes was determined using a color deconvolution algorithm on digital images. Two-sample, two-sided Wilcoxon tests were performed between indicated groups (*) $P < 0.05$; (**) $P < 0.01$. Center lines in box plots represent median values while lower and upper lines indicate the interquartile range (IQR; from the 25th to 75th percentile).

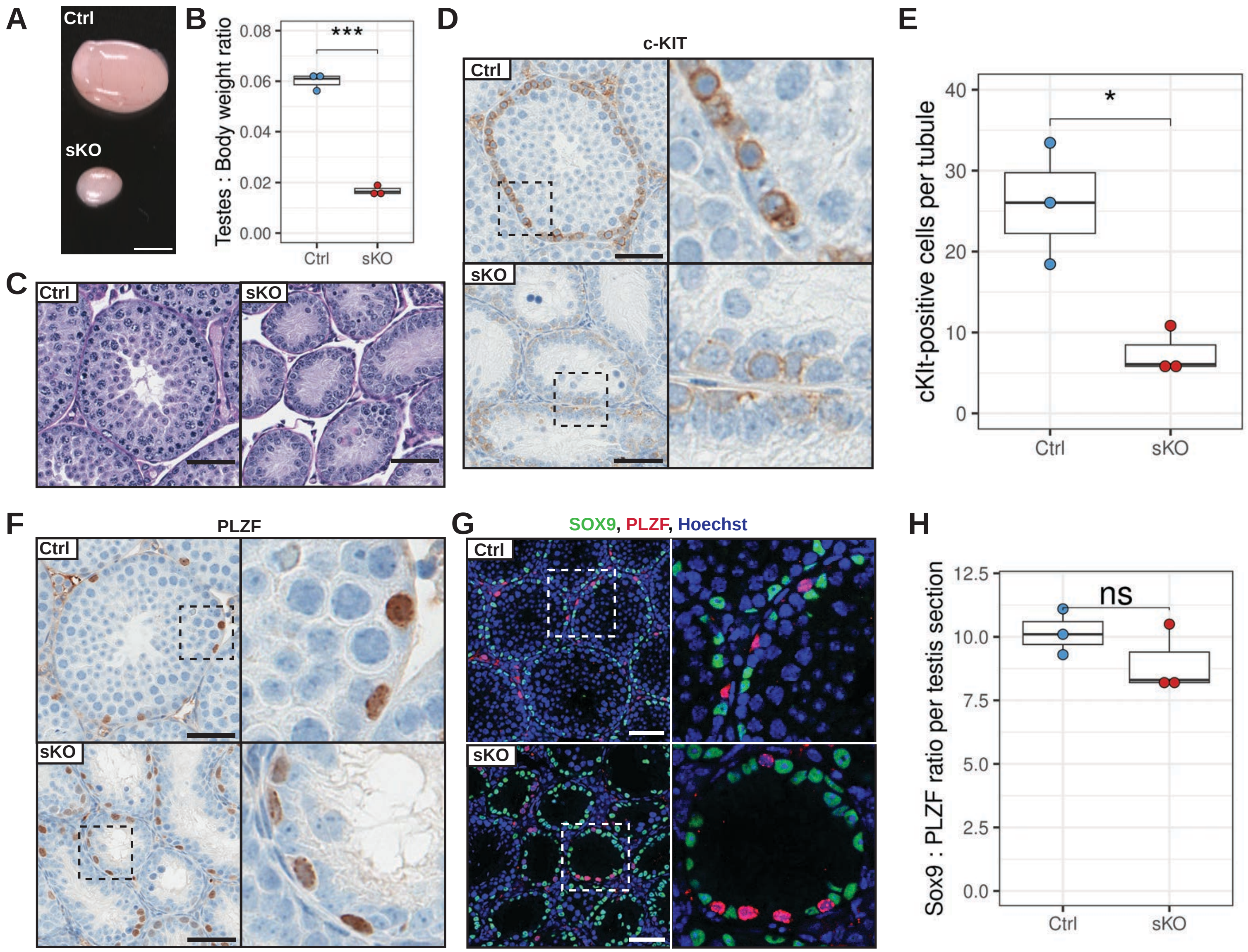
A
Ctrl
sKO
B
Testes : Body weight ratio
0.08
0.06
0.04
0.02
0.00
***
Ctrl
sKO
C
Ctrl
sKO
D
c-KIT
Ctrl
sKO
E
cKit-positive cells per tubule
40
30
20
10
0
*
Ctrl
sKO
F
PLZF
Ctrl
sKO
G
SOX9, PLZF, Hoechst
Ctrl
sKO
H
Sox9 : PLZF ratio per testis section
12.5
10.0
7.5
5.0
2.5
0.0
ns
Ctrl
sKO

Supplementary Figure 4. Ngn3-cre mediated *Kdm2a* deletion reduces c-KIT+ spermatogonia

(A) Testes of Ctrl and *Kdm2a* sKO (*Kdm2a*$^{2lox/2lox}$; *Ngn3*$^{cre/+}$) males. Scale bar = 2mm.

(B) Combined testes weights of Ctrl and sKO mice normalized to normalized to body weight, n = 3 males per genotype. Two-sample, two-sided Wilcoxon tests were performed between indicated groups ***$P \leq 0.001$. Center lines in box plots represent median values while lower and upper lines indicate the interquartile range (IQR; from the 25th to 75th percentile).

(C) PAS-stained testis sections from Ctrl and *Kdm2a* sKO mice at 26d (n = 3 males per genotype).

(D) Immunohistochemical staining of 26d testes from Ctrl (n=3) and *Kdm2a* sKO (n=3) males with an anti-c-KIT antibody and a hematoxylin counter stain. Boxed regions in left panels are magnified to the right. Scale bar = 50 µm.

(E) Number of c-KIT+ cells in Ctrl and sKO seminiferous tubules, 25 random tubules were evaluated from each animal. n = 3 per genotype, Two-sample, two-sided Wilcoxon tests were performed between indicated groups (“) $P< 0.05$. Center lines in box plots represent median values while lower and upper lines indicate the interquartile range (IQR; from the 25th to 75th percentile).

(F) Immunohistochemical staining of 26d testes from Ctrl (n=3) and *Kdm2a* sKO (n=3) males with an anti-PLZF antibody and hematoxylin counter stain. Boxed regions in left panels are magnified to the right. Scale bar = 50 µm.

(G) Immunofluorescence staining of testes from Ctrl (n=3) and sKO (n=3) using antibodies against PLZF (red) and SOX9 (Sertoli cell marker, green). Boxed regions in left panels are magnified to the right. Scale bar = 50 µm.

(H) Ratio of SOX9 and PLZF positive cells per testis section in Ctrl and sKO seminiferous tubules. Over 100 seminiferous tubules per mouse were analyzed. n = 3 males per group Two-sample, two-sided Wilcoxon tests were performed between indicated groups (ns) $P> 0.05$. Center lines in box plots represent median values while lower and upper lines indicate the interquartile range (IQR; from the 25th to 75th percentile).

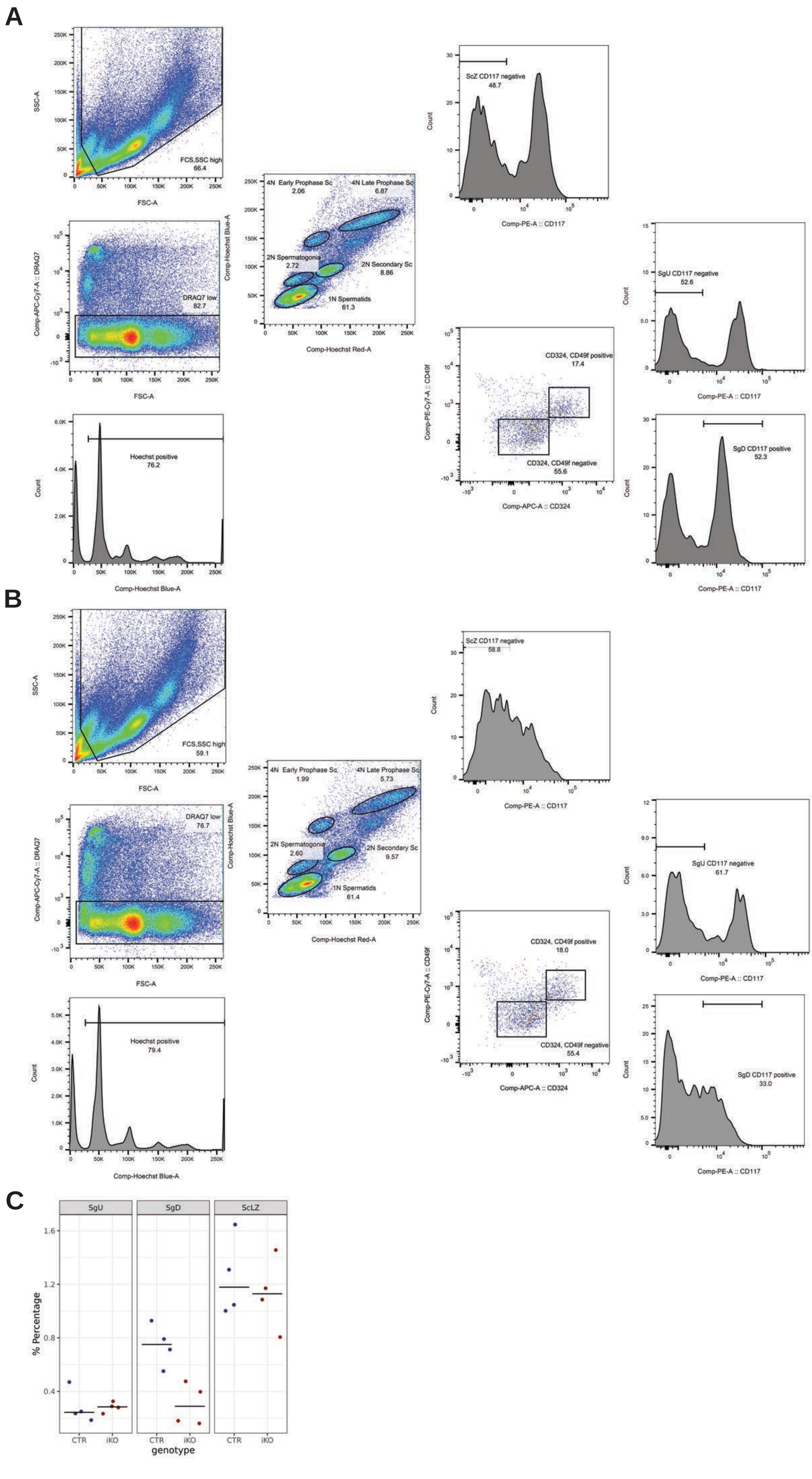
A
FCS,SSC high
66.4
SSC-A
FSC-A
DRAQ7 low
82.7
Comp-APC-Cy7-A :: DRAQ7
Hoechst positive
76.2
Count
Comp-Hoechst Blue-A
4N Early Prophase Sc
2.06
4N Late Prophase Sc
6.87
2N Spermatogonia
2.72
2N Secondary Sc
8.86
1N Spermatids
61.3
Comp-Hoechst Red-A
ScZ CD117 negative
48.7
Comp-PE-A :: CD117
CD324, CD49f positive
17.4
CD324, CD49f negative
55.6
Comp-PE-Cy7-A :: CD49f
Comp-APC-A :: CD324
SgU CD117 negative
52.6
SgD CD117 positive
52.3
B
FCS,SSC high
59.1
DRAQ7 low
76.7
Hoechst positive
79.4
4N Early Prophase Sc
1.99
4N Late Prophase Sc
5.73
2N Spermatogonia
2.60
2N Secondary Sc
9.57
1N Spermatids
61.4
ScZ CD117 negative
58.8
CD324, CD49f positive
18.0
CD324, CD49f negative
55.4
SgU CD117 negative
61.7
SgD CD117 positive
33.0
C
SgU
SgD
ScLZ
% Percentage
CTR
iKO
genotype

Supplementary Figure 5. FACS-based sorting of two populations of spermatogonia and early meiotic spermatocytes

(A) Representative FACS sorting profiles of germ cells with gating parameters from (A) Ctrl (*Kdm2a*$^{2lox/2lox}$) and (B) *Kdm2a* inducible knockout (iKO: *Kdm2a*$^{2lox/2lox}$; *CAGG-Cre-ER*^{TM}) at 10d post first tamoxifen treatment. FSC versus SSC plot indicating gate excluding the debris. FCS versus DRAQ7 plot indicating gate including viable cells. Hoechst-Blue histogram indicate gate with stained cells. Hoechst-Red versus Hoechst-Blue plot indicating gates containing populations of spermatogonia, meiotic spermatocytes and post-meiotic cells. CD324 (E-CADHERIN) versus CD49f (INTEGRIN ALPHA 6) plot showing gates containing undifferentiated spermatogonia SgU (CD324high, CD49fhigh) and differentiated spermatogonia SgD (CD324low, CD49flow). CD117 (c-KIT) histogram indicates the gate with SgU spermatogonia (Hoechst-Bluemid, Hoechst-Redlow, CD324high, CD49fhigh, CD117low). CD117 (c-KIT) histogram indicates the gate with SgD spermatogonia (Hoechst-Bluemid, Hoechst-Redlow, CD324low, CD49flow, CD117high).

(C) Dot plots representing the relative percentages of sorted SgU, SgD, and ScLZ (early meiotic prophase) to all viable Hoechst positive cells from Ctrl (n=4) and iKO (n=4) animals.

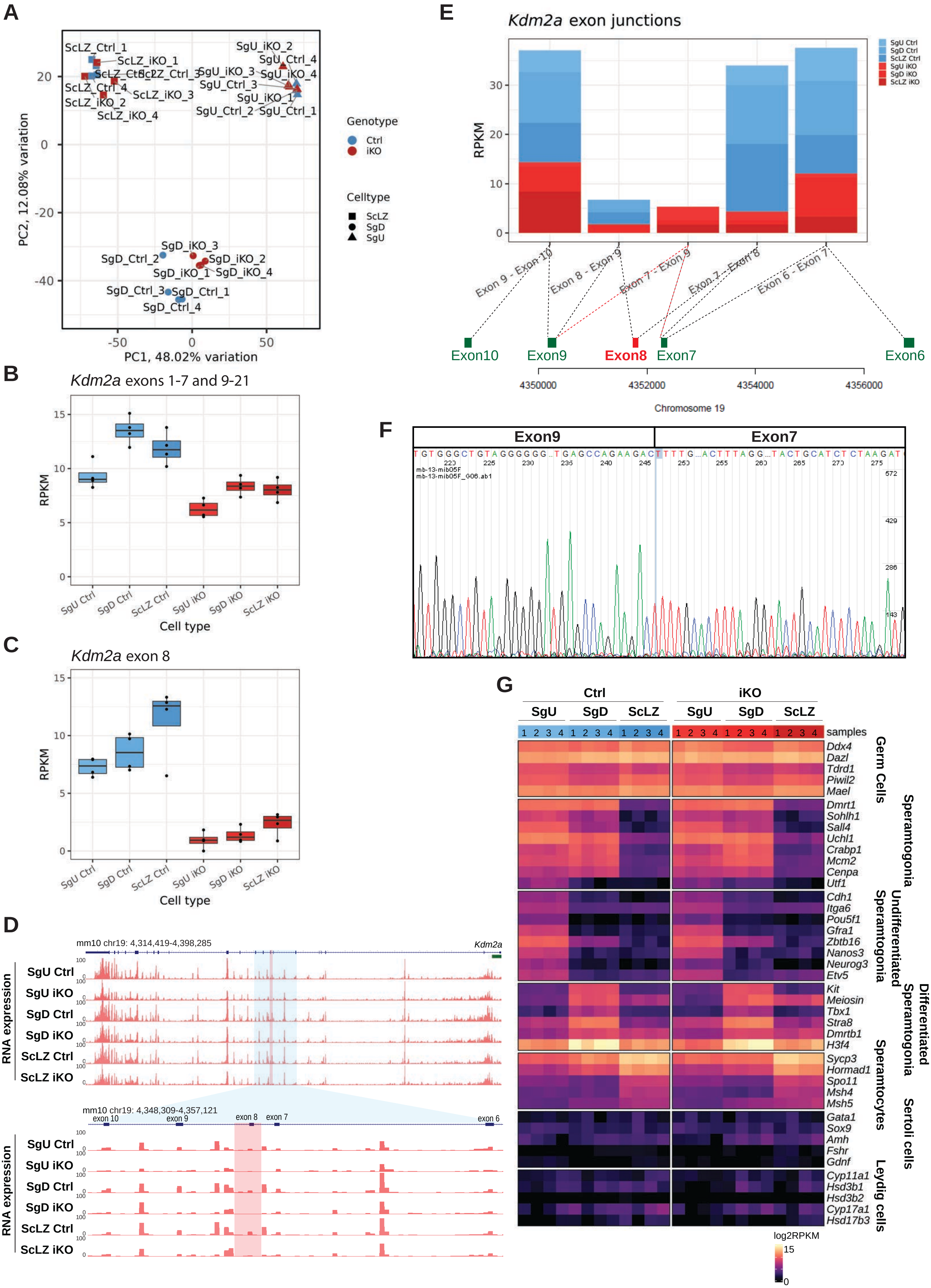

A
PC1, 48.02% variation
PC2, 12.08% variation
Genotype
Ctrl
iKO
Celltype
ScLZ
SgD
SgU
B
Kdm2a exons 1-7 and 9-21
RPKM
Cell type
C
Kdm2a exon 8
D
mm10 chr19: 4,314,419-4,398,285
Kdm2a
RNA expression
SgU Ctrl
SgU iKO
SgD Ctrl
SgD iKO
ScLZ Ctrl
ScLZ iKO
mm10 chr19: 4,348,309-4,357,121
exon 10
exon 9
exon 8
exon 7
exon 6
E
Kdm2a exon junctions
Exon 9 - Exon 10
Exon 8 - Exon 9
Exon 7 - Exon 9
Exon 7 - Exon 8
Exon 6 - Exon 7
Exon10
Exon9
Exon8
Exon7
Exon6
Chromosome 19
F
Exon9
Exon7
G
Ctrl
iKO
SgU
SgD
ScLZ
samples
Germ Cells
Spermatogonia
Undifferentiated Spermatogonia
Differentiated Spermatogonia
Spermatocytes
Sertoli cells
Leydig cells
Ddx4
Dazl
Tdrd1
Piwil2
Mael
Dmrt1
Sohlh1
Sall4
Uchl1
Crabp1
Mcm2
Cenpa
Utf1
Cdh1
Itga6
Pou5f1
Gfra1
Zbtb16
Nanos3
Neurog3
Etv5
Kit
Meiosin
Tbx1
Stra8
Dmrtb1
H3f4
Sycp3
Hormad1
Spo11
Msh4
Msh5
Gata1
Sox9
Amh
Fshr
Gdnf
Cyp11a1
Hsd3b1
Hsd3b2
Cyp17a1
Hsd17b3
log2RPKM

Supplementary Figure 6. Reduced expression across the entire Kdm2a locus in iKO cells. (A) Principal component analysis of Ctrl (*Kdm2a*$^{2lox/2lox}$) (n=4) and *Kdm2a* inducible knockout (iKO: *Kdm2a*$^{2lox/2lox}$; *CAGG-Cre-ER*^{TM}) (n=4) at 10d post first tamoxifen treatment RNA-seq samples based on gene expression.

(B) Boxplots showing the normalized expression values for individual samples displayed as datapoints of RNA-seq reads mapping to the *Kdm2a* exons 1-7 and 9-21 in Ctrl and *Kdm2a* iKO samples.

(C) Boxplots showing the normalized expression values for individual samples displayed as datapoints of RNA-seq reads mapping to the *Kdm2a* exon 8 (the lox site flanking exon) in Ctrl and *Kdm2a* iKO samples.

(D) UCSC genome browser snapshot of the *Kdm2a* locus (upper) and zoomed up to exons 6 to 10 (lower), showing tracks for RNA expression (red) from SgU, SgD and ScLZ Ctrl (*Kdm2a*$^{2lox/2lox}$) and *Kdm2a* inducible knockout (iKO: *Kdm2a*$^{2lox/2lox}$; *CAGG-Cre-ER*^{TM}) (n=4 for each genotype) animals 10d post first tamoxifen treatment. The floxed region containing exon 8 is highlighted in red.

(E) Barplot showing abundance of RNA-seq reads mapping to exon-exon junctions (dashed black lines) around exon 8, in Ctrl and *Kdm2a* iKO samples. Heights of the bars are indicative of the number of reads. Dashed red line denotes the presence of atypical exon7-exon9 junction in *Kdm2a* iKO samples only.

(F) Sanger sequencing of RT-PCR products identified exon 7 - exon 9 spanning sequences in 9d post first tamoxifen treatment *Kdm2a* iKO (*Kdm2a*$^{2lox/2lox}$; *Actb*$^{CreERT2/+}$) testes.

(G) Heatmap showing the $\log_2$ RPKM values of selected cell type gene markers in SgU and SgD from Ctrl and *Kdm2a* iKO samples.

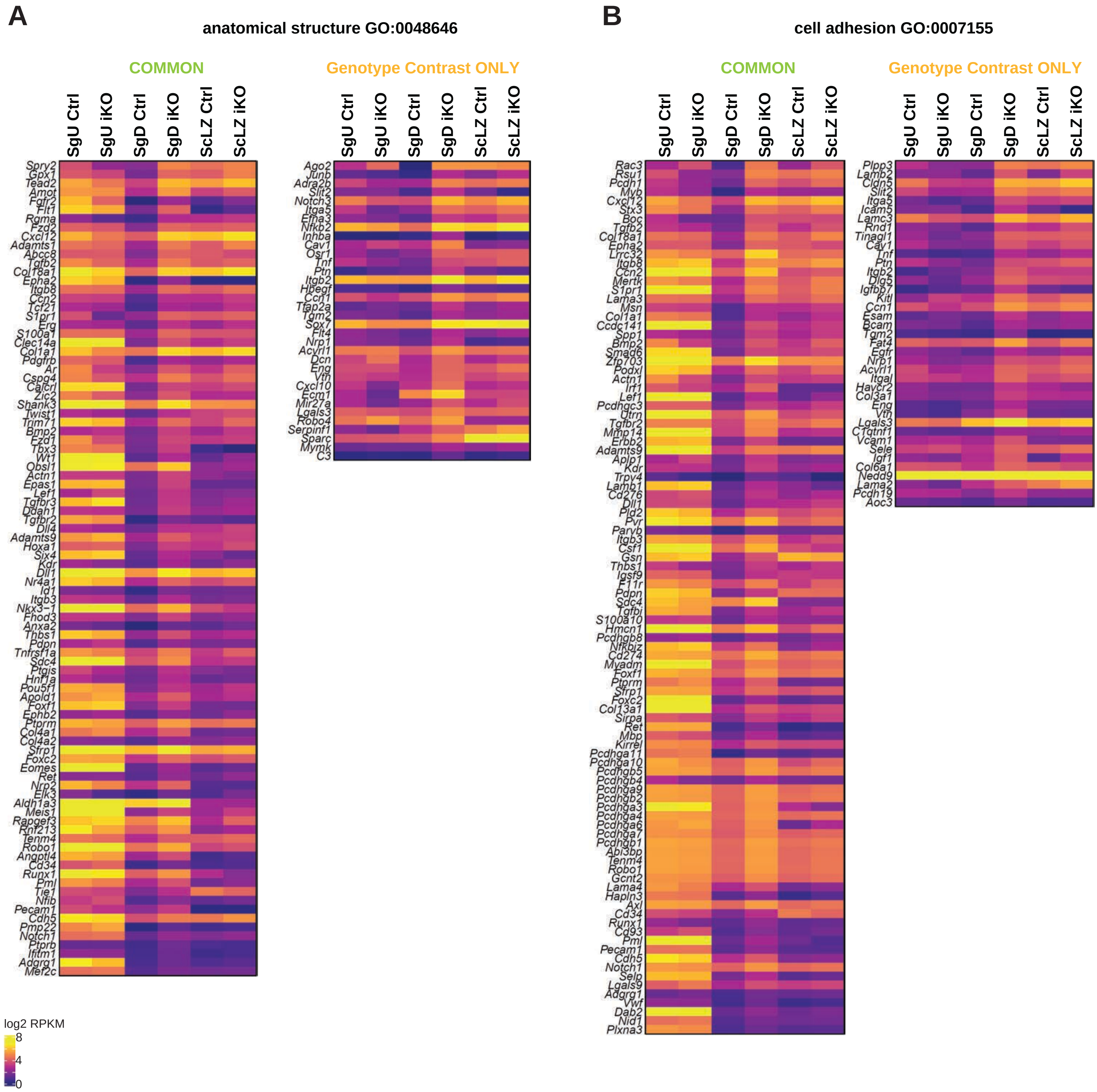

A
anatomical structure GO:0048646
COMMON
Genotype Contrast ONLY
SgU Ctrl
SgU iKO
SgD Ctrl
SgD iKO
ScLZ Ctrl
ScLZ iKO
Spry2 Gpx1 Tead2 Amot Fgfr2 Flt1 Rgma Fzd2 Cxcl12 Adamts1 Abcc8 Tgfb2 Col18a1 Epha2 Itgb8 Ccn2 Tcf21 S1pr1 Erg S100a1 Clec14a Col1a1 Pdgfrb Ar Cspg4 Calcrl Zic2 Shank3 Twist1 Trim71 Bmp2 Fzd1 Tbx3 Wt1 Obsl1 Actn1 Epas1 Lef1 Tgfbr3 Ddah1 Tgfbr2 Dll4 Adamts9 Hoxa1 Six4 Kdr Dll1 Nr4a1 Id1 Itgb3 Nkx3-1 Fhod3 Anxa2 Thbs1 Pdpn Tnfrsf1a Sdc4 Ptgis Hnf1a Pou5f1 Apold1 Foxf1 Ephb2 Ptprm Col4a1 Col4a2 Sfrp1 Foxc2 Eomes Ret Nrp2 Elk3 Aldh1a3 Meis1 Rapgef3 Rnf213 Tenm4 Robo1 Angptl4 Cd34 Runx1 Pml Tie1 Nfib Pecam1 Cdh5 Pmp22 Notch1 Ptprb Ifitm1 Adgrg1 Mef2c
Ago2 Junb Adra2b Slit2 Notch3 Itga5 Efna3 Nfkb2 Inhba Cav1 Osr1 Tnf Ptn Itgb2 Hbegf Ccn1 Tfap2a Tgm2 Sox7 Flt4 Nrp1 Acvrl1 Dcn Eng Vtn Cxcl10 Ecm1 Mir27a Lgals3 Robo4 Serpinf1 Sparc Mymk C3
log2 RPKM
8
4
0
B
cell adhesion GO:0007155
COMMON
Genotype Contrast ONLY
SgU Ctrl
SgU iKO
SgD Ctrl
SgD iKO
ScLZ Ctrl
ScLZ iKO
Rac3 Rsu1 Pcdh1 Myb Cxcl12 Stx3 Boc Tgfb2 Col18a1 Epha2 Lrrc32 Itgb8 Ccn2 Mertk S1pr1 Lama3 Msn Col1a1 Ccdc141 Spp1 Bmp2 Smad6 Zfp703 Podxl Actn1 Irf1 Lef1 Pcdhgc3 Utrn Tgfbr2 Mmp14 Erbb2 Adamts9 Aplp1 Kdr Trpv4 Lamb1 Cd276 Dll1 Pld2 Pvr Parvb Itgb3 Csf1 Gsn Thbs1 Igsf9 F11r Pdpn Sdc4 Tgfbi S100a10 Hmcn1 Pcdhgb8 Nfkbiz Cd274 Myadm Foxf1 Ptprm Sfrp1 Foxc2 Col13a1 Sirpa Ret Mbp Kirrel Pcdhga11 Pcdhga10 Pcdhgb5 Pcdhgb4 Pcdhga9 Pcdhgb2 Pcdhga3 Pcdhga4 Pcdhga6 Pcdhga7 Pcdhgb1 Abi3bp Tenm4 Robo1 Gcnt2 Lama4 Hapln3 Axl Cd34 Runx1 Cd93 Pml Pecam1 Cdh5 Notch1 Selp Lgals9 Adgrg1 Vwf Dab2 Nid1 Plxna3
Plpp3 Lamb2 Cldn5 Slit2 Itga5 Icam5 Lamc3 Rnd1 Tinagl1 Cav1 Tnf Ptn Itgb2 Dlg5 Igfbp7 Kitl Ccn1 Esam Bcam Tgm2 Fat4 Egfr Nrp1 Acvrl1 Itgal Havcr2 Col3a1 Eng Vtn Lgals3 C1qtnf1 Vcam1 Sele Igf1 Col6a1 Nedd9 Lama2 Pcdh19 Aoc3

C

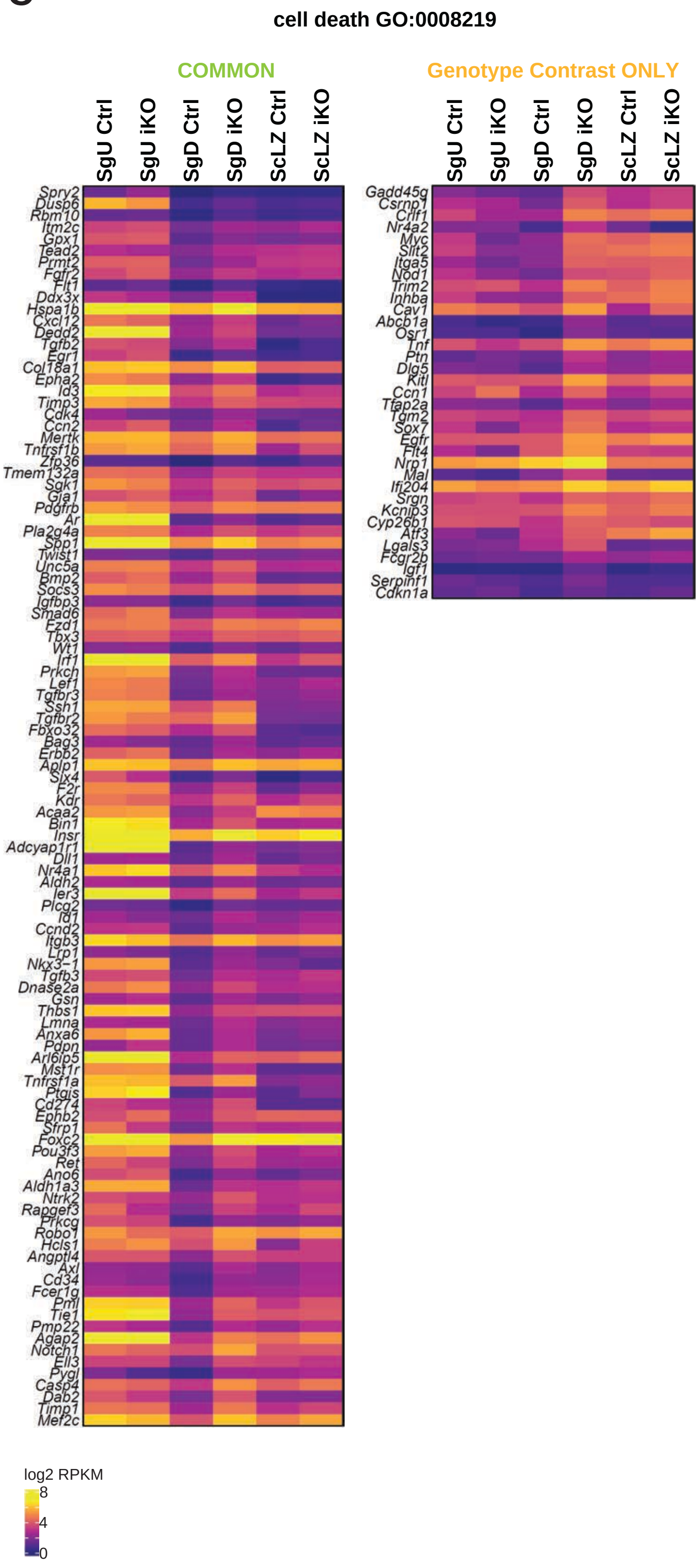
cell death GO:0008219
COMMON
Genotype Contrast ONLY
SgU Ctrl
SgU iKO
SgD Ctrl
SgD iKO
ScLZ Ctrl
ScLZ iKO
Spry2
Dusp6
Rbm10
Itm2c
Gpx1
Tead2
Prmt2
Fgf2
Flt1
Ddx3x
Hspa1b
Cxcl12
Dedd2
Tgfb2
Egr1
Col18a1
Epha2
Id3
Timp3
Cdk4
Ccn2
Mertk
Tnfrsf1b
Zfp36
Tmem132a
Sgk1
Gja1
Pdgfrb
Ar
Pla2g4a
Spp1
Twist1
Unc5a
Bmp2
Socs3
Igfbp3
Smad6
Fzd1
Tbx3
Wt1
Irf1
Prkch
Lef1
Tgfbr3
Ssh1
Tgfbr2
Fbxo32
Bag3
Erbb2
Aplp1
Six4
F2r
Kdr
Acaa2
Bin1
Insr
Adcyap1r1
Dll1
Nr4a1
Aldh2
Ier3
Plcg2
Id1
Ccnd2
Itgb3
Lrp1
Nkx3-1
Tgfb3
Dnase2a
Gsn
Thbs1
Lmna
Anxa6
Pdpn
Arl6ip5
Mst1r
Tnfrsf1a
Ptgis
Cd274
Ephb2
Sfrp1
Foxc2
Pou3f3
Ret
Ano6
Aldh1a3
Ntrk2
Rapgef3
Prkcq
Robo1
Hcls1
Angptl4
Axl
Cd34
Fcer1g
Pml
Tie1
Pmp22
Agap2
Notch1
Ell3
Pygl
Casp4
Dab2
Timp1
Mef2c
Gadd45g
Csrnp1
Crlf1
Nr4a2
Myc
Slit2
Itga5
Nod1
Trim2
Inhba
Cav1
Abcb1a
Osr1
Tnf
Ptn
Dlg5
Kitl
Ccn1
Tfap2a
Tgm2
Sox7
Egfr
Flt4
Nrp1
Mal
Ifi204
Srgn
Kcnip3
Cyp26b1
Atf3
Lgals3
Fcgr2b
Igf1
Serpinf1
Cdkn1a
log2 RPKM
8
4
0

D

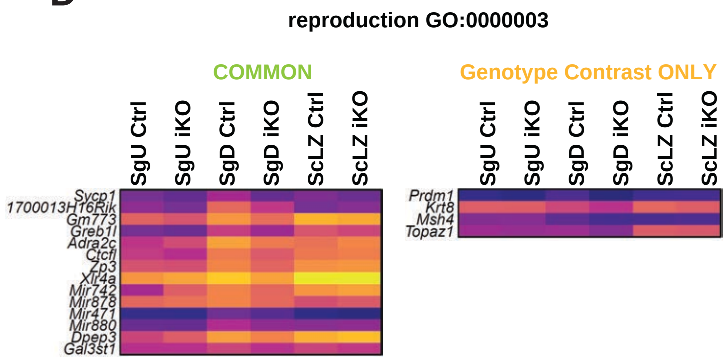
reproduction GO:0000003
COMMON
Genotype Contrast ONLY
SgU Ctrl
SgU iKO
SgD Ctrl
SgD iKO
ScLZ Ctrl
ScLZ iKO
Sycp1
1700013H16Rik
Gm773
Greb1l
Adra2c
Ctcfl
Zp3
Xlr4a
Mir742
Mir878
Mir471
Mir880
Dpep3
Gal3st1
Prdm1
Krt8
Msh4
Topaz1

Supplementary Figure 7. Gene ontology analysis of genes differentially expressed in *Kdm2a* iKO germ cells.

(A-D) Heatmap showing the $\log_2$ RPKM values of genes belonging to GO slim terms (A) anatomical structure GO:0048646, (B) cell adhesion GO:0007155, (C) cell death GO:0008219 and (D) reproduction GO:0000003 and are significantly misregulated in either both the developmental contrast (Ctrl SgD versus Ctrl SgU) and genotype contrast (iKO SgD versus Ctrl SgD) [COMMON] or significantly misregulated only in genotype contrast (iKO SgD versus Ctrl SgD) [Genotype Contrast ONLY].

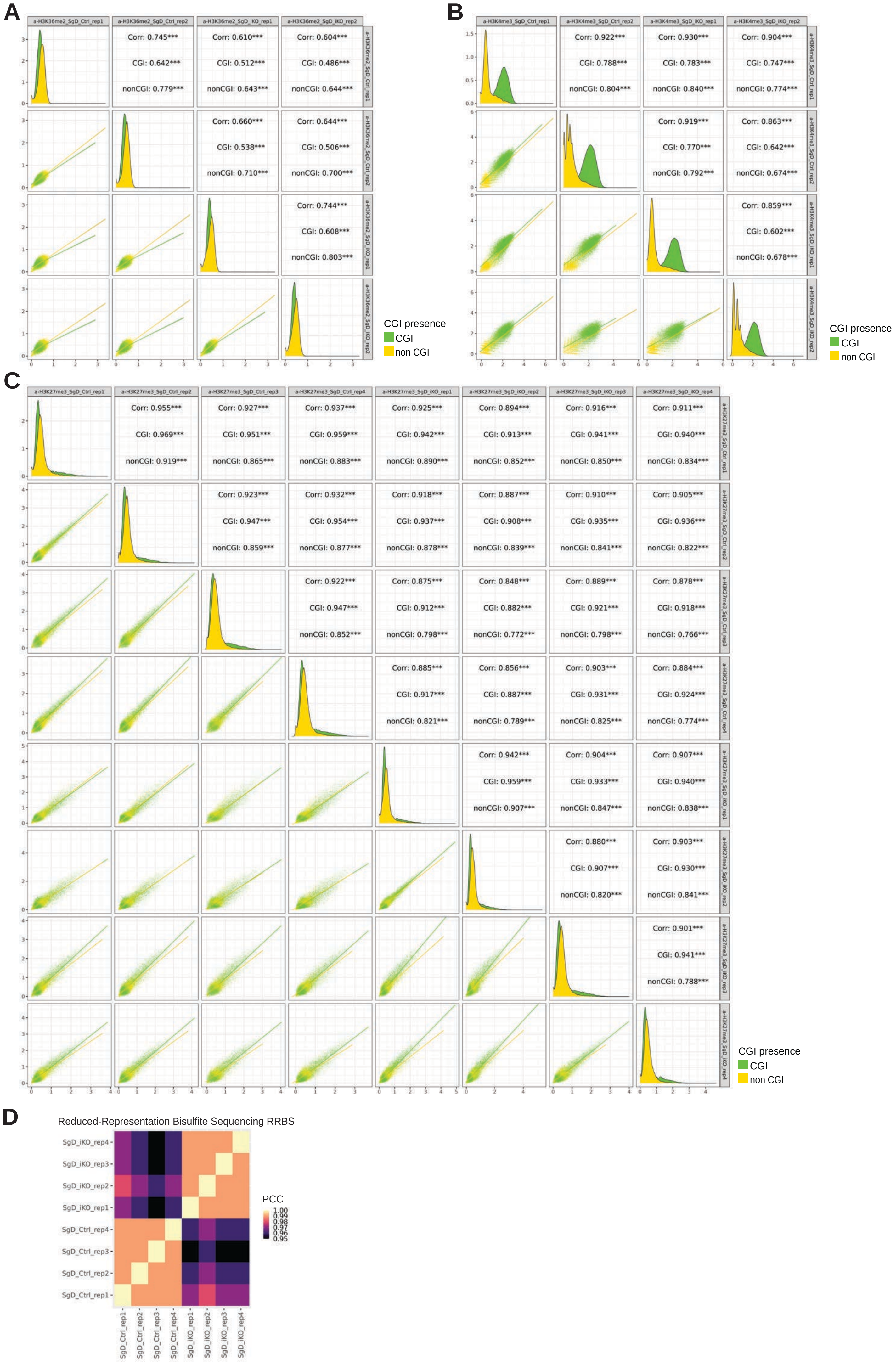
A
a-H3K36me2_SgD_Ctrl_rep1
a-H3K36me2_SgD_Ctrl_rep2
a-H3K36me2_SgD_iKO_rep1
a-H3K36me2_SgD_iKO_rep2
Corr: 0.745***
CGI: 0.642***
nonCGI: 0.779***
Corr: 0.610***
CGI: 0.512***
nonCGI: 0.643***
Corr: 0.604***
CGI: 0.486***
nonCGI: 0.644***
Corr: 0.660***
CGI: 0.538***
nonCGI: 0.710***
Corr: 0.644***
CGI: 0.506***
nonCGI: 0.700***
Corr: 0.744***
CGI: 0.608***
nonCGI: 0.803***
CGI presence
CGI
non CGI
B
a-H3K4me3_SgD_Ctrl_rep1
a-H3K4me3_SgD_Ctrl_rep2
a-H3K4me3_SgD_iKO_rep1
a-H3K4me3_SgD_iKO_rep2
Corr: 0.922***
CGI: 0.788***
nonCGI: 0.804***
Corr: 0.930***
CGI: 0.783***
nonCGI: 0.840***
Corr: 0.904***
CGI: 0.747***
nonCGI: 0.774***
Corr: 0.919***
CGI: 0.770***
nonCGI: 0.792***
Corr: 0.863***
CGI: 0.642***
nonCGI: 0.674***
Corr: 0.859***
CGI: 0.602***
nonCGI: 0.678***
CGI presence
CGI
non CGI
C
a-H3K27me3_SgD_Ctrl_rep1
a-H3K27me3_SgD_Ctrl_rep2
a-H3K27me3_SgD_Ctrl_rep3
a-H3K27me3_SgD_Ctrl_rep4
a-H3K27me3_SgD_iKO_rep1
a-H3K27me3_SgD_iKO_rep2
a-H3K27me3_SgD_iKO_rep3
a-H3K27me3_SgD_iKO_rep4
Corr: 0.955***
CGI: 0.969***
nonCGI: 0.919***
Corr: 0.927***
CGI: 0.951***
nonCGI: 0.865***
Corr: 0.937***
CGI: 0.959***
nonCGI: 0.883***
Corr: 0.925***
CGI: 0.942***
nonCGI: 0.890***
Corr: 0.894***
CGI: 0.913***
nonCGI: 0.852***
Corr: 0.916***
CGI: 0.941***
nonCGI: 0.850***
Corr: 0.911***
CGI: 0.940***
nonCGI: 0.834***
Corr: 0.923***
CGI: 0.947***
nonCGI: 0.859***
Corr: 0.932***
CGI: 0.954***
nonCGI: 0.877***
Corr: 0.918***
CGI: 0.937***
nonCGI: 0.878***
Corr: 0.887***
CGI: 0.908***
nonCGI: 0.839***
Corr: 0.910***
CGI: 0.935***
nonCGI: 0.841***
Corr: 0.905***
CGI: 0.936***
nonCGI: 0.822***
Corr: 0.922***
CGI: 0.947***
nonCGI: 0.852***
Corr: 0.875***
CGI: 0.912***
nonCGI: 0.798***
Corr: 0.848***
CGI: 0.882***
nonCGI: 0.772***
Corr: 0.889***
CGI: 0.921***
nonCGI: 0.798***
Corr: 0.878***
CGI: 0.918***
nonCGI: 0.766***
Corr: 0.885***
CGI: 0.917***
nonCGI: 0.821***
Corr: 0.856***
CGI: 0.887***
nonCGI: 0.789***
Corr: 0.903***
CGI: 0.931***
nonCGI: 0.825***
Corr: 0.884***
CGI: 0.924***
nonCGI: 0.774***
Corr: 0.942***
CGI: 0.959***
nonCGI: 0.907***
Corr: 0.904***
CGI: 0.933***
nonCGI: 0.847***
Corr: 0.907***
CGI: 0.940***
nonCGI: 0.838***
Corr: 0.880***
CGI: 0.907***
nonCGI: 0.820***
Corr: 0.903***
CGI: 0.930***
nonCGI: 0.841***
Corr: 0.901***
CGI: 0.941***
nonCGI: 0.788***
CGI presence
CGI
non CGI
D
Reduced-Representation Bisulfite Sequencing RRBS
SgD_iKO_rep4
SgD_iKO_rep3
SgD_iKO_rep2
SgD_iKO_rep1
SgD_Ctrl_rep4
SgD_Ctrl_rep3
SgD_Ctrl_rep2
SgD_Ctrl_rep1
PCC
1.00
0.99
0.98
0.97
0.96
0.95

E

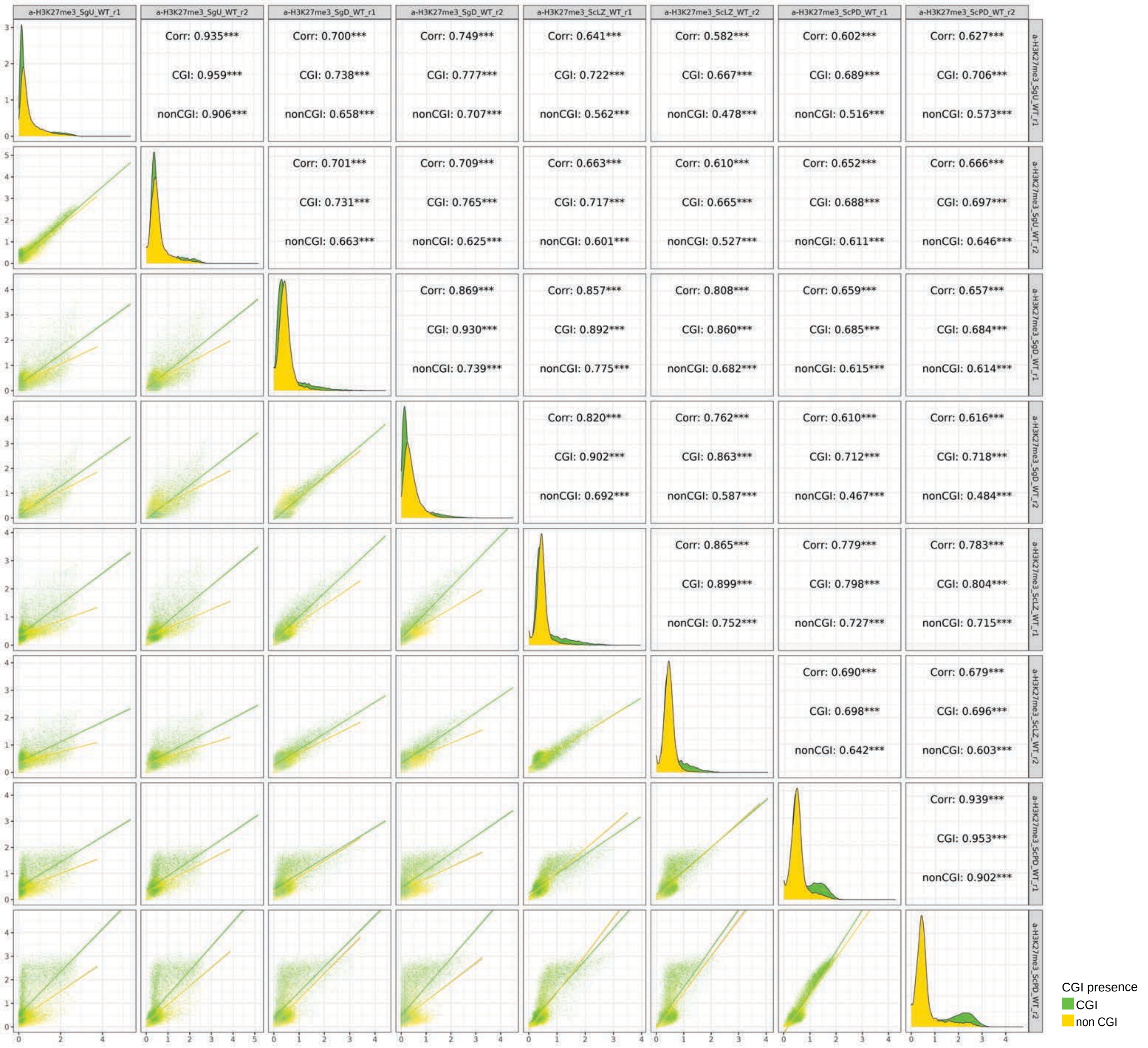
a-H3K27me3_SgU_WT_r1
a-H3K27me3_SgU_WT_r2
a-H3K27me3_SgD_WT_r1
a-H3K27me3_SgD_WT_r2
a-H3K27me3_ScLZ_WT_r1
a-H3K27me3_ScLZ_WT_r2
a-H3K27me3_ScPD_WT_r1
a-H3K27me3_ScPD_WT_r2
Corr: 0.935*** CGI: 0.959*** nonCGI: 0.906***
Corr: 0.700*** CGI: 0.738*** nonCGI: 0.658***
Corr: 0.749*** CGI: 0.777*** nonCGI: 0.707***
Corr: 0.641*** CGI: 0.722*** nonCGI: 0.562***
Corr: 0.582*** CGI: 0.667*** nonCGI: 0.478***
Corr: 0.602*** CGI: 0.689*** nonCGI: 0.516***
Corr: 0.627*** CGI: 0.706*** nonCGI: 0.573***
Corr: 0.701*** CGI: 0.731*** nonCGI: 0.663***
Corr: 0.709*** CGI: 0.765*** nonCGI: 0.625***
Corr: 0.663*** CGI: 0.717*** nonCGI: 0.601***
Corr: 0.610*** CGI: 0.665*** nonCGI: 0.527***
Corr: 0.652*** CGI: 0.688*** nonCGI: 0.611***
Corr: 0.666*** CGI: 0.697*** nonCGI: 0.646***
Corr: 0.869*** CGI: 0.930*** nonCGI: 0.739***
Corr: 0.857*** CGI: 0.892*** nonCGI: 0.775***
Corr: 0.808*** CGI: 0.860*** nonCGI: 0.682***
Corr: 0.659*** CGI: 0.685*** nonCGI: 0.615***
Corr: 0.657*** CGI: 0.684*** nonCGI: 0.614***
Corr: 0.820*** CGI: 0.902*** nonCGI: 0.692***
Corr: 0.762*** CGI: 0.863*** nonCGI: 0.587***
Corr: 0.610*** CGI: 0.712*** nonCGI: 0.467***
Corr: 0.616*** CGI: 0.718*** nonCGI: 0.484***
Corr: 0.865*** CGI: 0.899*** nonCGI: 0.752***
Corr: 0.779*** CGI: 0.798*** nonCGI: 0.727***
Corr: 0.783*** CGI: 0.804*** nonCGI: 0.715***
Corr: 0.690*** CGI: 0.698*** nonCGI: 0.642***
Corr: 0.679*** CGI: 0.696*** nonCGI: 0.603***
Corr: 0.939*** CGI: 0.953*** nonCGI: 0.902***
CGI presence
CGI
non CGI

Supplementary Figure 8. Chromatin analysis at gene transcriptional start sites (TSS) +/- 1kb genomic windows.

(A) Scatterplots [left side] show the H3K36me2 $\log_2$ RPKM values at gene TSS +/- 1kb genomic windows detected by ULI-NChIP-seq between pairs of biological replicated samples of differentiated spermatogonia (SgD) from Ctrl (*Kdm2a*$^{2lox/2lox}$) (n=2) and *Kdm2a* inducible knockout (iKO: *Kdm2a*$^{2lox/2lox}$; *CAGG-Cre-ER*^{TM}) (n=2) animals 10d post first tamoxifen treatment. The diagonal line plots show the distribution of the H3K36me2 $\log_2$ RPKM values in each replicate. The Pearson correlation coefficient between indicated pairs of biological replicates is shown in the upper right. The gene TSSs which contain a CpG island are highlighted in green and the gene TSS without a CpG island are highlighted in yellow.

(B) Scatterplots [left side] show the H3K4me3 $\log_2$ RPKM values at gene TSS +/- 1kb genomic windows detected by ULI-NChIP-seq between pairs of biological replicated samples of differentiated spermatogonia (SgD) from Ctrl (*Kdm2a*$^{2lox/2lox}$) (n=2) and *Kdm2a* inducible knockout (iKO: *Kdm2a*$^{2lox/2lox}$; *CAGG-Cre-ER*^{TM}) (n=2) animals 10d post first tamoxifen treatment. The diagonal line plots show the distribution of the H3K4me3 $\log_2$ RPKM values in each replicate. The Pearson correlation coefficient between indicated pairs of biological replicates is shown in the upper right. The gene TSSs which contain a CpG island are highlighted in green and the gene TSS without a CpG island are highlighted in yellow.

(C) Scatterplots [left side] show the H3K27me3 $\log_2$ RPKM values at gene TSS (Transcriptional Start Site) +/- 1kb genomic windows detected by ULI-NChIP-seq between pairs of biological replicated samples of differentiated spermatogonia (SgD) from Ctrl (*Kdm2a*$^{2lox/2lox}$) (n=4) and *Kdm2a* inducible knockout (iKO: *Kdm2a*$^{2lox/2lox}$; *CAGG-Cre-ER*^{TM}) (n=4) animals 10d post first tamoxifen treatment. The diagonal line plots show the distribution of the H3K27me3 $\log_2$ RPKM values in each replicate. The Pearson correlation coefficient between indicated pairs of biological replicates is shown in the upper right. The gene TSSs which contain a CpG island are highlighted in green and the gene TSS without a CpG island are highlighted in yellow.

(D) Heatmap showing the Pearson correlation coefficient of percentage DNA methylation at gene TSS +/- 1kb genomic windows detected by RRBS between pairs of biological replicated

samples of differentiated spermatogonia (SgD) from Ctrl (*Kdm2a*$^{2lox/2lox}$) (n=4) and *Kdm2a* inducible knockout (iKO: *Kdm2a*$^{2lox/2lox}$; *CAGG-Cre-ER*^{TM}) (n=4) animals.

(E) Scatterplots [left side] show the H3K27me3 log_2 RPKM values at gene TSS +/- 1kb genomic windows detected by ULI-NChIP-seq between pairs of biological replicated samples of Undifferentiated spermatogonia (SgU), Differentiated spermatogonia (SgD), Leptotene/Zygotene spermatocytes (ScLZ) and Pachytene/Diplotene spermatocytes (ScPD) from WT (*Kdm2a*$^{+/+}$) (n=2) animals. The diagonal line plots show the distribution of the H3K27me3 log_2 RPKM values in each replicate. The Pearson correlation coefficient between indicated pairs of biological replicates is shown in the upper right. The gene TSSs which contain a CpG island are highlighted in green and the gene TSS without a CpG island are highlighted in yellow.

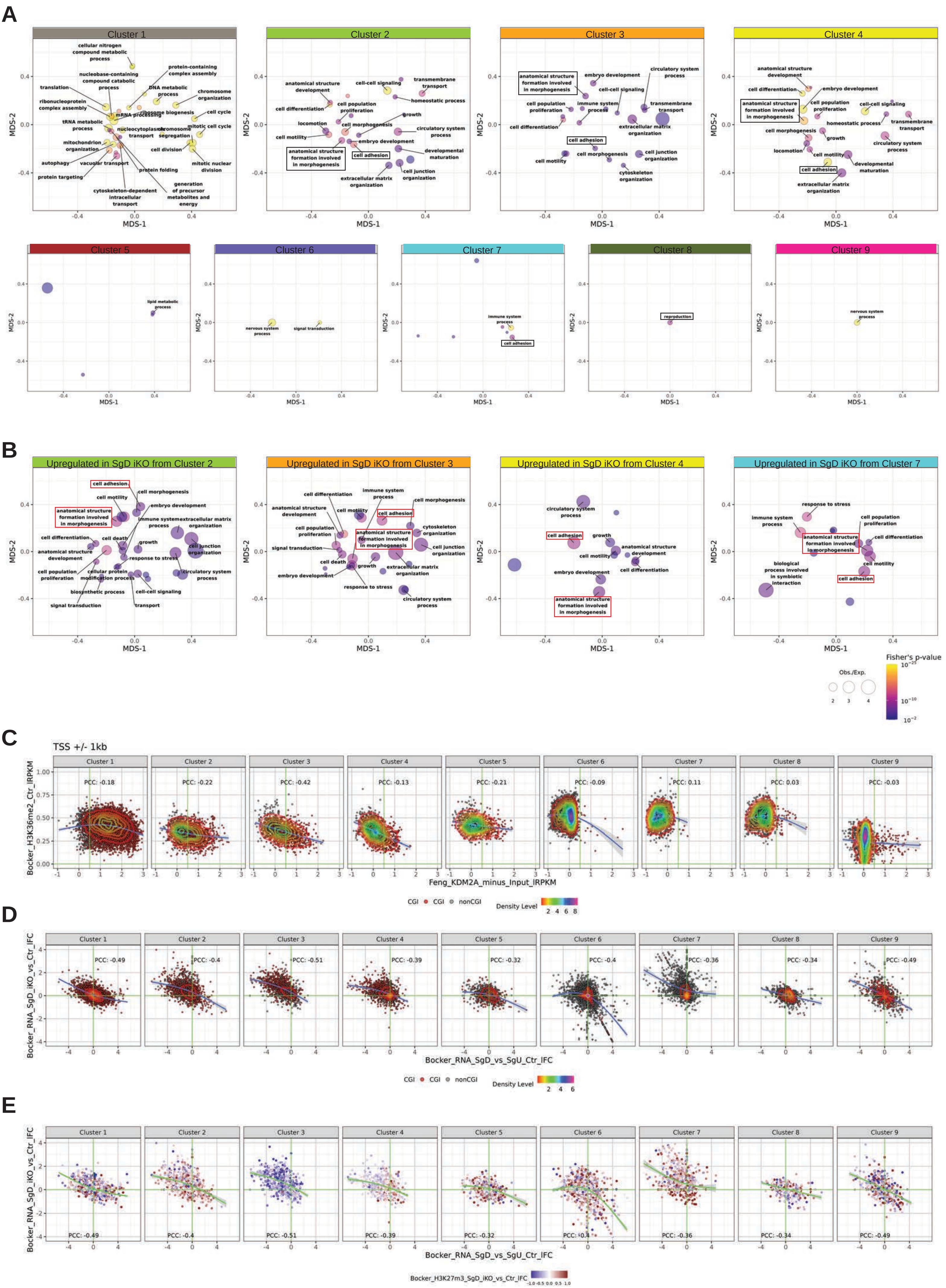
A
Cluster 1
Cluster 2
Cluster 3
Cluster 4
Cluster 5
Cluster 6
Cluster 7
Cluster 8
Cluster 9
MDS-1
MDS-2
cellular nitrogen compound metabolic process
nucleobase-containing compound catabolic process
protein-containing complex assembly
translation
DNA metabolic process
chromosome organization
ribonucleoprotein complex assembly
mRNA processing
ribosome biogenesis
cell cycle
tRNA metabolic process
nucleocytoplasmic transport
chromosome segregation
mitotic cell cycle
mitochondrion organization
cell division
autophagy
vacuolar transport
protein folding
mitotic nuclear division
protein targeting
cytoskeleton-dependent intracellular transport
generation of precursor metabolites and energy
anatomical structure development
cell-cell signaling
transmembrane transport
homeostatic process
cell differentiation
cell population proliferation
growth
locomotion
cell morphogenesis
circulatory system process
cell motility
embryo development
anatomical structure formation involved in morphogenesis
cell adhesion
developmental maturation
cell junction organization
extracellular matrix organization
immune system process
cytoskeleton organization
lipid metabolic process
nervous system process
signal transduction
reproduction
B
Upregulated in SgD iKO from Cluster 2
Upregulated in SgD iKO from Cluster 3
Upregulated in SgD iKO from Cluster 4
Upregulated in SgD iKO from Cluster 7
cell death
response to stress
cellular protein modification process
biosynthetic process
transport
biological process involved in symbiotic interaction
Fisher's p-value
Obs./Exp.
C
TSS +/- 1kb
Bocker_H3K36me2_Ctr_lRPKM
Feng_KDM2A_minus_Input_lRPKM
PCC: -0.18
PCC: -0.22
PCC: -0.42
PCC: -0.13
PCC: -0.21
PCC: -0.09
PCC: 0.11
PCC: 0.03
PCC: -0.03
CGI ● CGI ● nonCGI
Density Level
D
Bocker_RNA_SgD_iKO_vs_Ctr_lFC
Bocker_RNA_SgD_vs_SgU_Ctr_lFC
PCC: -0.49
PCC: -0.4
PCC: -0.51
PCC: -0.39
PCC: -0.32
PCC: -0.4
PCC: -0.36
PCC: -0.34
PCC: -0.49
E
Bocker_H3K27m3_SgD_iKO_vs_Ctr_lFC

F

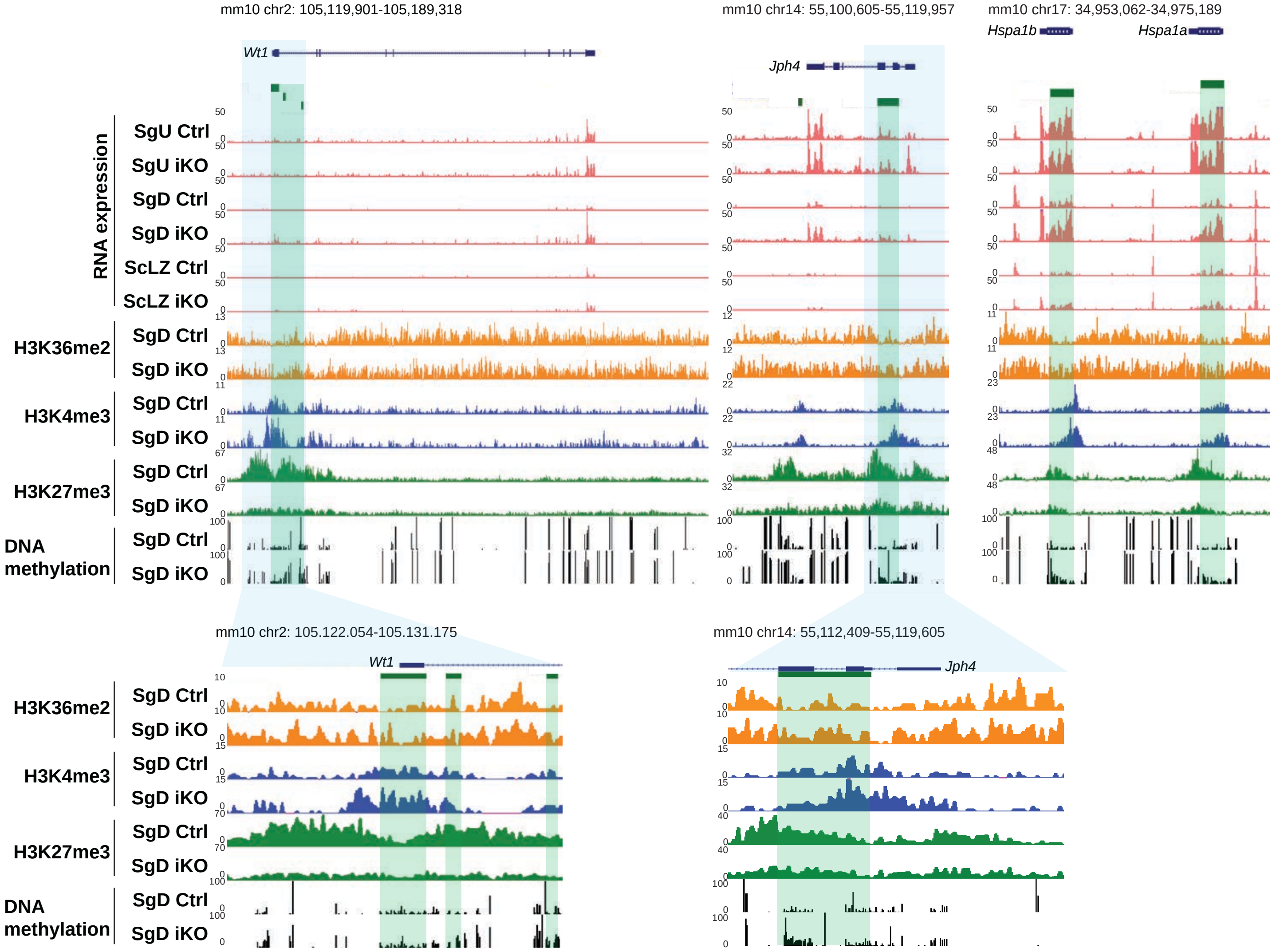
mm10 chr2: 105,119,901-105,189,318
mm10 chr14: 55,100,605-55,119,957
mm10 chr17: 34,953,062-34,975,189
Wt1
Jph4
Hspa1b
Hspa1a
RNA expression
SgU Ctrl
SgU iKO
SgD Ctrl
SgD iKO
ScLZ Ctrl
ScLZ iKO
H3K36me2
H3K4me3
H3K27me3
DNA methylation
mm10 chr2: 105.122.054-105.131.175
mm10 chr14: 55,112,409-55,119,605

Supplementary Figure 9. Chromatin signature and functional annotation of KDM2A target genes.

(A) Bubble plots showing significantly enriched GOslim terms associated with genes belonging to clusters 1-9 as defined in Figure 5. The size of the bubble denotes enrichment and color denotes significance. Distance of the bubbles centers represents similarity of genes contained in each GOslim term. GOslim terms overrepresented in *Kdm2a* iKO SgD upregulated genes are highlighted with boxes.

(B) Bubble plots showing significantly enriched GOslim terms associated with genes belonging to clusters 2,3,4 and 7 and being significantly upregulated in *Kdm2a* iKO SgD. The size of the bubble denotes enrichment and color denotes significance. Distance of the bubbles centers represents similarity of genes contained in each GOslim term. GOslim terms appearing common in all clusters are highlighted with boxes.

(C) Scatter plots showing the KDM2A normalized to Input enrichment from Feng *et al.* (28) (x-axis) plotted against the H3K36me2 enrichment in Ctrl SgD (y-axis) at gene TSS +/-1kb belonging to clusters 1 to 9 as defined in Figure 5. CGI-containing TSSs are marked in red. The color scale of the contour denotes the density of the individual points. Blue lines represent loess weighted linearly regressed data. The Pearson correlation coefficient (PCC) between x and y variables is reported.

(D) Scatter plots showing expression fold-changes between Ctrl SgD versus Ctrl SgU (developmental contrast, x-axis) compared to those of iKO SgD versus Ctrl SgD (genotype contrast, y-axis) for genes belonging to clusters 1 to 9 as defined in Figure 5. CGI-containing TSSs are marked in red. The color scale of the contour denotes the density of the individual points. Blue lines represent loess weighted linearly regressed data. The PCC between x and y variables is reported.

(E) Scatter plots showing the gene expression fold-changes between Ctrl SgD versus Ctrl SgU (developmental contrast, x-axis) compared to those of iKO SgD versus Ctrl SgD (genotype contrast, y-axis) for genes belonging to clusters 1 to 9 as defined in Figure 5. The color scale of individual points represents H3K27me3 enrichment fold-changes among iKO SgD versus

Ctrl SgD. Green lines represent loess weighted linearly regressed data. The PCC between x and y variables is reported.

(F) UCSC genome browser snapshot of the *Wt1* (left), *Jph4* (middle) and *Hspa1a* - *Hspa1b* (right) loci, showing tracks for RNA expression (red) from SgU, SgD and ScLZ Ctrl (*Kdm2a*$^{2lox/2lox}$) and *Kdm2a* inducible knockout (iKO: *Kdm2a*$^{2lox/2lox}$; *CAGG-Cre-ER*^{TM}) (n=4 for each genotype) animals 10d post first tamoxifen treatment. Tracks for H3K36me2 (orange), H3K4me3 (blue), H3K27me3 (green) occupancies assessed by ULI-NChIP-seq in Ctrl and iKO SgD (n=2 for H3K36me2 and H3K4me3, n=4 for H3K27me3 for each genotype) 10d post first tamoxifen treatment. Tracks for DNA methylation percentage (black) assessed by RRBS in Ctrl and iKO SgD (n=4 for each genotype) 10d post first tamoxifen treatment. The promoter associated CGIs are highlighted in green.

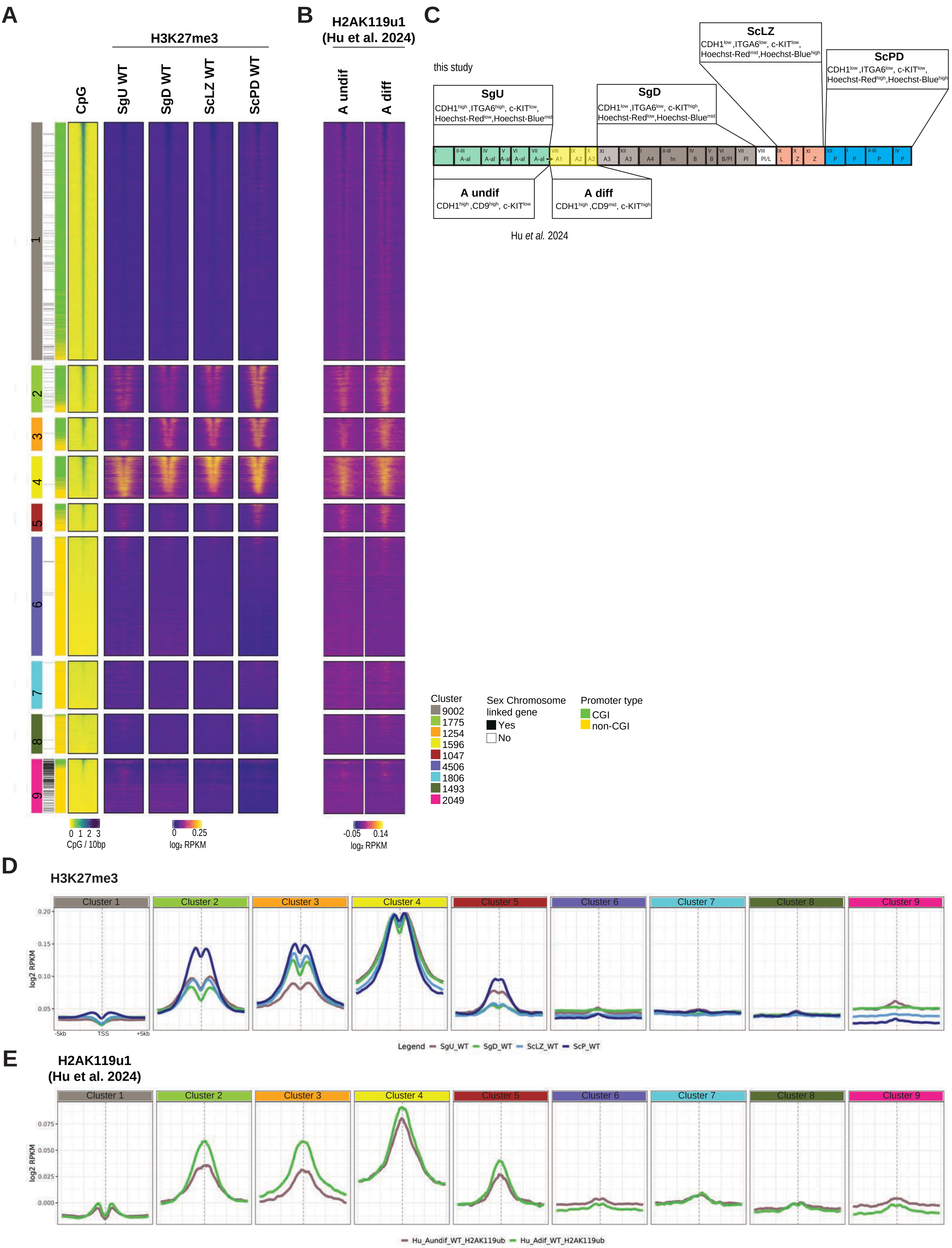
A
H3K27me3
CpG
SgU WT
SgD WT
ScLZ WT
ScPD WT
1
2
3
4
5
6
7
8
9
0 1 2 3
CpG / 10bp
0 0.25
log2 RPKM
B
H2AK119u1
(Hu et al. 2024)
A undif
A diff
-0.05 0.14
log2 RPKM
C
this study
SgU
CDH1high,ITGA6high, c-KITlow,
Hoechst-Redlow,Hoechst-Bluemid
SgD
CDH1low,ITGA6low, c-KIThigh,
Hoechst-Redlow,Hoechst-Bluemid
ScLZ
CDH1low,ITGA6low, c-KITlow,
Hoechst-Redmid,Hoechst-Bluehigh
ScPD
CDH1low,ITGA6low, c-KITlow,
Hoechst-Redhigh,Hoechst-Bluehigh
A undif
CDH1high,CD9high, c-KITlow
A diff
CDH1high,CD9mid, c-KIThigh
Hu et al. 2024
Cluster
9002
1775
1254
1596
1047
4506
1806
1493
2049
Sex Chromosome linked gene
Yes
No
Promoter type
CGI
non-CGI
D
H3K27me3
Cluster 1
Cluster 2
Cluster 3
Cluster 4
Cluster 5
Cluster 6
Cluster 7
Cluster 8
Cluster 9
log2 RPKM
-5kb
TSS
+5kb
Legend SgU_WT SgD_WT ScLZ_WT ScP_WT
E
H2AK119u1
(Hu et al. 2024)
Hu_Aundif_WT_H2AK119ub Hu_Adif_WT_H2AK119ub

Supplementary Figure 10. Dynamics of H3K27me3 and H2AK119ub1 during spermatogenesis.

A) Heatmap showing H3K27me3 of 5kb regions upstream/downstream of their corresponding TSSs. All genes were classified into 9 clusters as defined in Figure 5A. From left to right: Cluster identity, Chromosomal location of the gene, Presence or absence of CpG island from the gene promoter, CpG density at TSS +/- 5kb, $\log_2$ RPKM values of H3K27me3 ULI-NChIP-seq for non-overlapping 50bp genomic window located +/- 5kb from TSS from SgU (Undifferentiated Spermatogonia), SgD (Differentiated Spermatogonia), ScLZ (Leptotene/Zygotene Spermatocytes) and ScPD (Pachytene/Diplotene Spermatocytes) WT samples (n=2 for each celltype).

B) Heatmap showing H2AK119u1 of 5kb regions upstream/downstream relative to their TSSs. All genes were classified into 9 clusters shown in Figure 5A. From left to right: log2RPKM values of H2AK119u1 Cut&Tag for non-overlapping 50bp genomic windows located +/- 5kb from TSS in undifferentiated and differentiated type A spermatogonia from Hu et al., 2024 (29).

(C) Scheme showing FACS sorting antigens (21) to isolate populations of (Undifferentiated Spermatogonia), SgD (Differentiated Spermatogonia), ScLZ (Leptotene/Zygotene Spermatocytes) and ScPD (Pachytene/Diplotene Spermatocytes) in our study (upper panels), and type A undifferentiated and type A differentiated spermatogonia from Hu *et al.,* 2024 (29).

D) Line plots showing average H3K27me3 $\log_2$RPKM values at TSSs +/- 5kb belonging to the clusters 1-9 as defined in Figure 5 and assessed by ULI-NChIP-seq from SgU, SgD, ScLZ and ScPD WT samples (n=2 for each celltype).

E) Line plots showing average H2AK119u1 $\log_2$RPKM values at TSSs +/- 5kb belonging to the clusters 1-9 as defined in Figure 5 and assessed by Cut &Tag in undifferentiated and differentiated type A spermatogonia from Hu et al., 2024 (29).

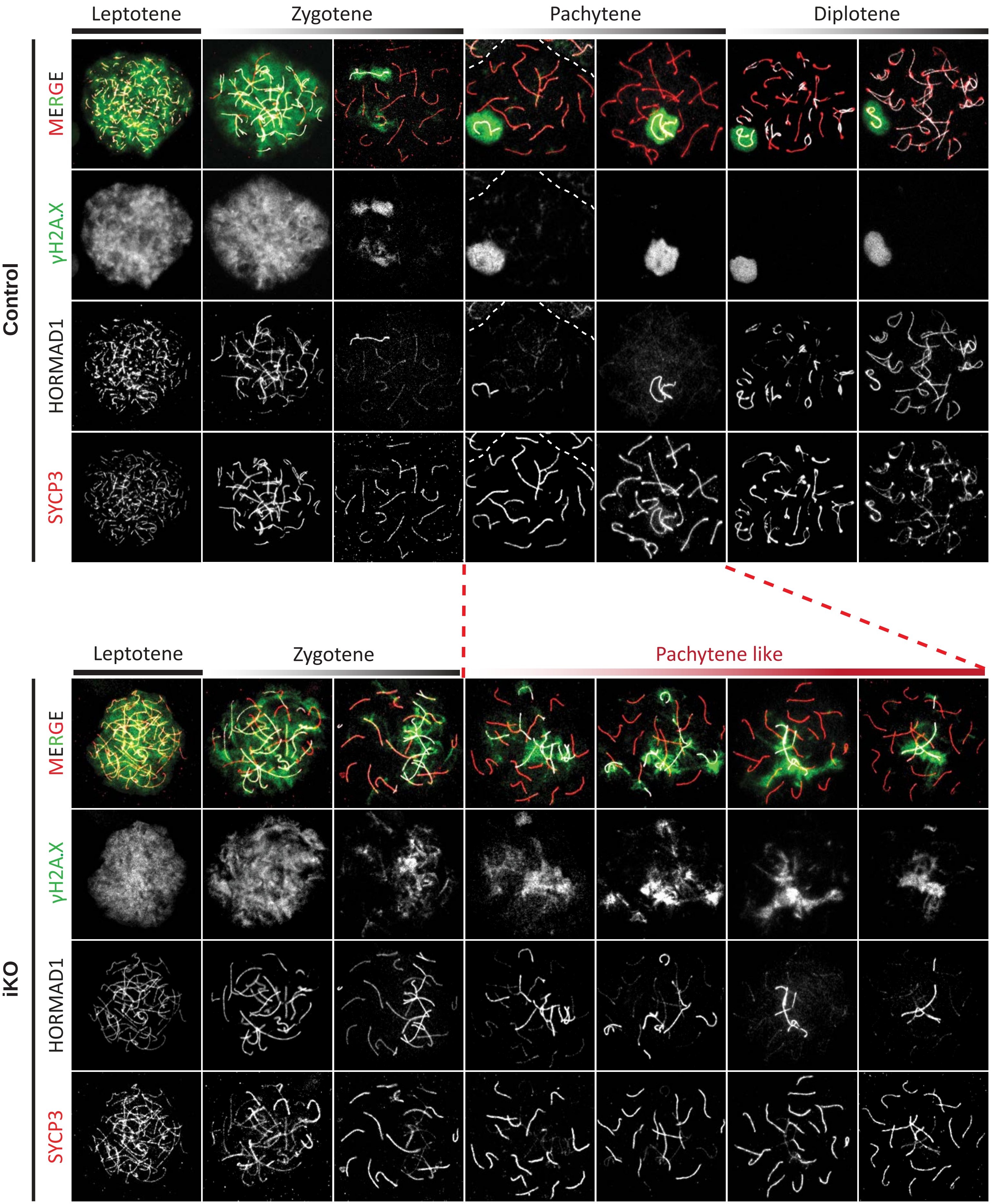
Leptotene
Zygotene
Pachytene
Diplotene
Control
MERGE
γH2A.X
HORMAD1
SYCP3
Leptotene
Zygotene
Pachytene like
iKO
MERGE
γH2A.X
HORMAD1
SYCP3

Supplementary Figure 11. Impaired meiotic progression in *Kdm2a* iKO spermatocytes

Immunofluorescence images of drying-down spreads of spermatocytes at different stages of meiotic prophase from Ctrl (*Kdm2a*$^{2lox/2lox}$) (n=3) and *Kdm2a* inducible knockout (iKO: *Kdm2a*$^{2lox/2lox}$; *Actb*$^{CreERT2/+}$) (n=3) at 9d post first tamoxifen treatment co-stained with anti-γH2A.X, anti-SYCP3, and anti-HORMAD1 antibodies as indicated. The “pachytene like” label indicates *Kdm2a* iKO spermatocytes that do not complete synapsis.

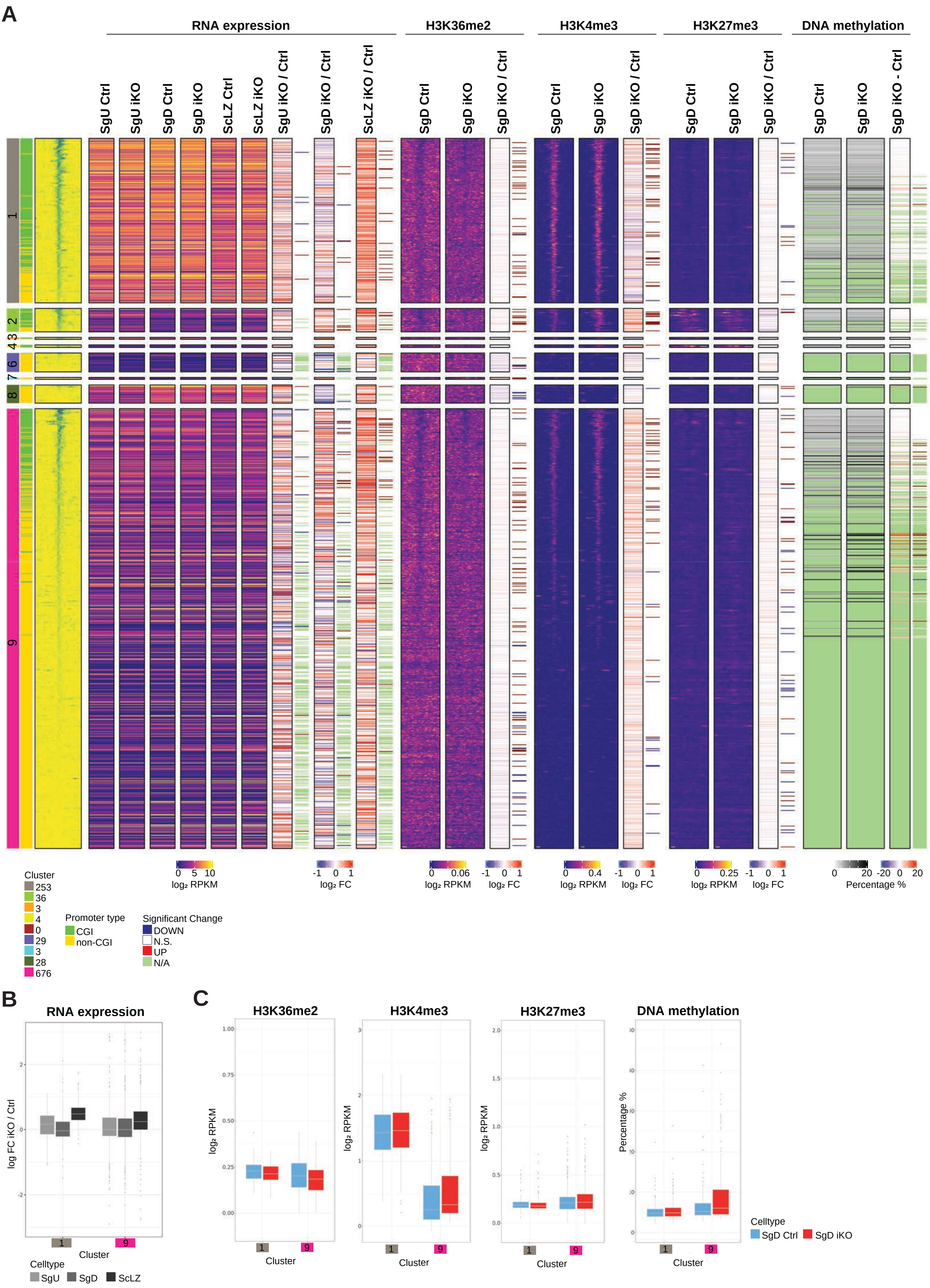
A
RNA expression
H3K36me2
H3K4me3
H3K27me3
DNA methylation
SgU Ctrl
SgU iKO
SgD Ctrl
SgD iKO
ScLZ Ctrl
ScLZ iKO
SgU iKO / Ctrl
SgD iKO / Ctrl
ScLZ iKO / Ctrl
SgD Ctrl
SgD iKO
SgD iKO / Ctrl
SgD iKO - Ctrl
1
2
3
4
6
7
8
9
0 5 10
log₂ RPKM
-1 0 1
log₂ FC
0 0.06
0 0.4
0 0.25
0 20 -20 0 20
Percentage %
Cluster
253
36
3
4
0
29
3
28
676
Promoter type
CGI
non-CGI
Significant Change
DOWN
N.S.
UP
N/A
B
RNA expression
log FC iKO / Ctrl
2
0
-2
1
9
Cluster
Celltype
SgU
SgD
ScLZ
C
H3K36me2
H3K4me3
H3K27me3
DNA methylation
log₂ RPKM
1.00
0.75
0.50
0.25
0.00
3
2
1
0
2.0
1.5
1.0
0.5
0.0
Percentage %
1
9
Cluster
Celltype
SgD Ctrl
SgD iKO

Supplementary Figure 12. Chromosome X linked genes are upregulated in *Kdm2a* iKO Leptotene-Zygotene spermatocytes

A) Heatmap of X-linked genes grouped according to clusters defined in Figure 5A.

B) Boxplot showing RNA expression lFC for X chromosome located genes belonging to clusters 1 and 9 of *Kdm2a* iKO over Ctrl SgU, SgD and ScLZ.

C) Boxplot showing a-H3K36me2, a-H3K4me3 and a-H3K27me3 ULI-NChIP-seq $\log_2$ RPKM values and DNA methylation percentages at TSS +/- 1kb of X chromosome located genes belonging to clusters 1 and 9 of Ctrl and *Kdm2a* iKO SgD samples.

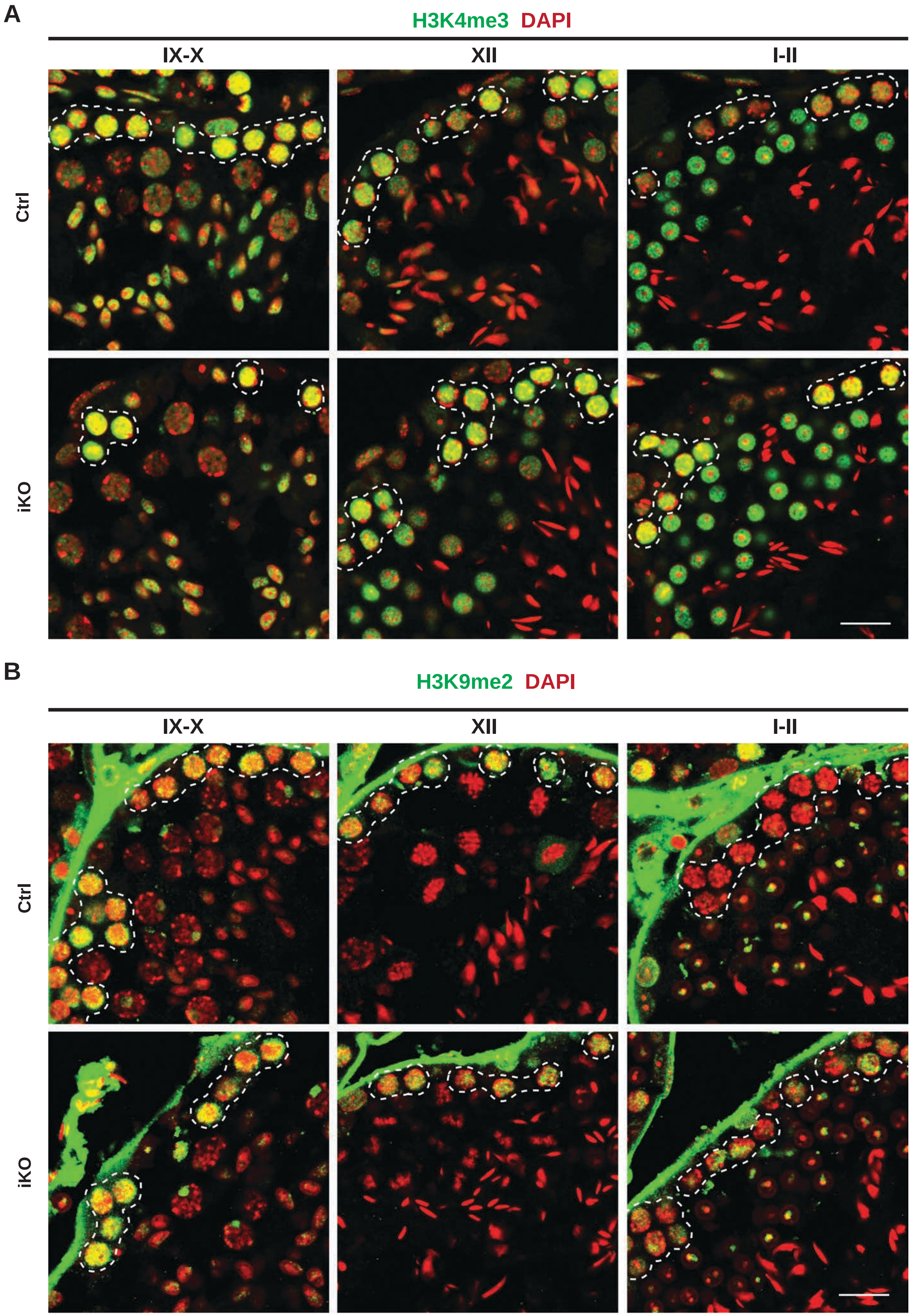
A
H3K4me3 DAPI
IX-X
XII
I-II
Ctrl
iKO
B
H3K9me2 DAPI
IX-X
XII
I-II
Ctrl
iKO

Supplementary Figure 13. *Kdm2a* iKO pachytene-like spermatocytes fail to reduce H3K4me3 and H3K9me2.

(A-B) Immunofluorescence stainings of testis sections from Ctrl (*Kdm2a*$^{2lox/2lox}$) (n=3) and *Kdm2a* inducible knockout (iKO: *Kdm2a*$^{2lox/2lox}$; *Actb*$^{CreERT2/+}$) (n=3) at 9d post first tamoxifen treatment, stained with (A) anti-H3K4me3 and (B) anti-H3K9me2 antibodies. Nuclei were stained with DAPI (red). Dashed lines highlight early meiotic cells (leptotene (IX-X), zygotene (XI-XII), and early pachytene(-like) (I-III) stages). Scale bars = 10μm.

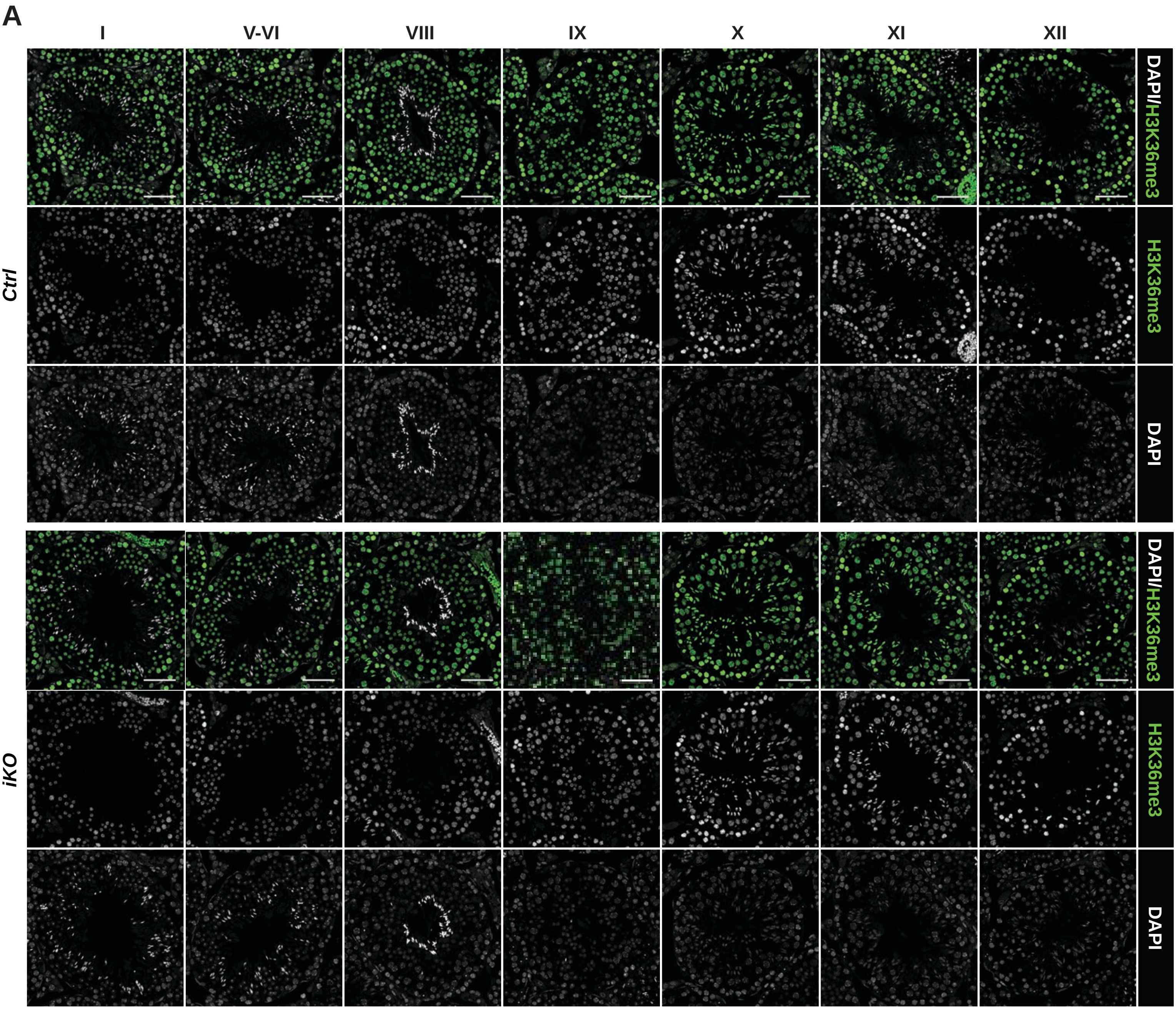

A
I
V-VI
VIII
IX
X
XI
XII
DAPI/H3K36me3
H3K36me3
DAPI
Ctrl
iKO

B

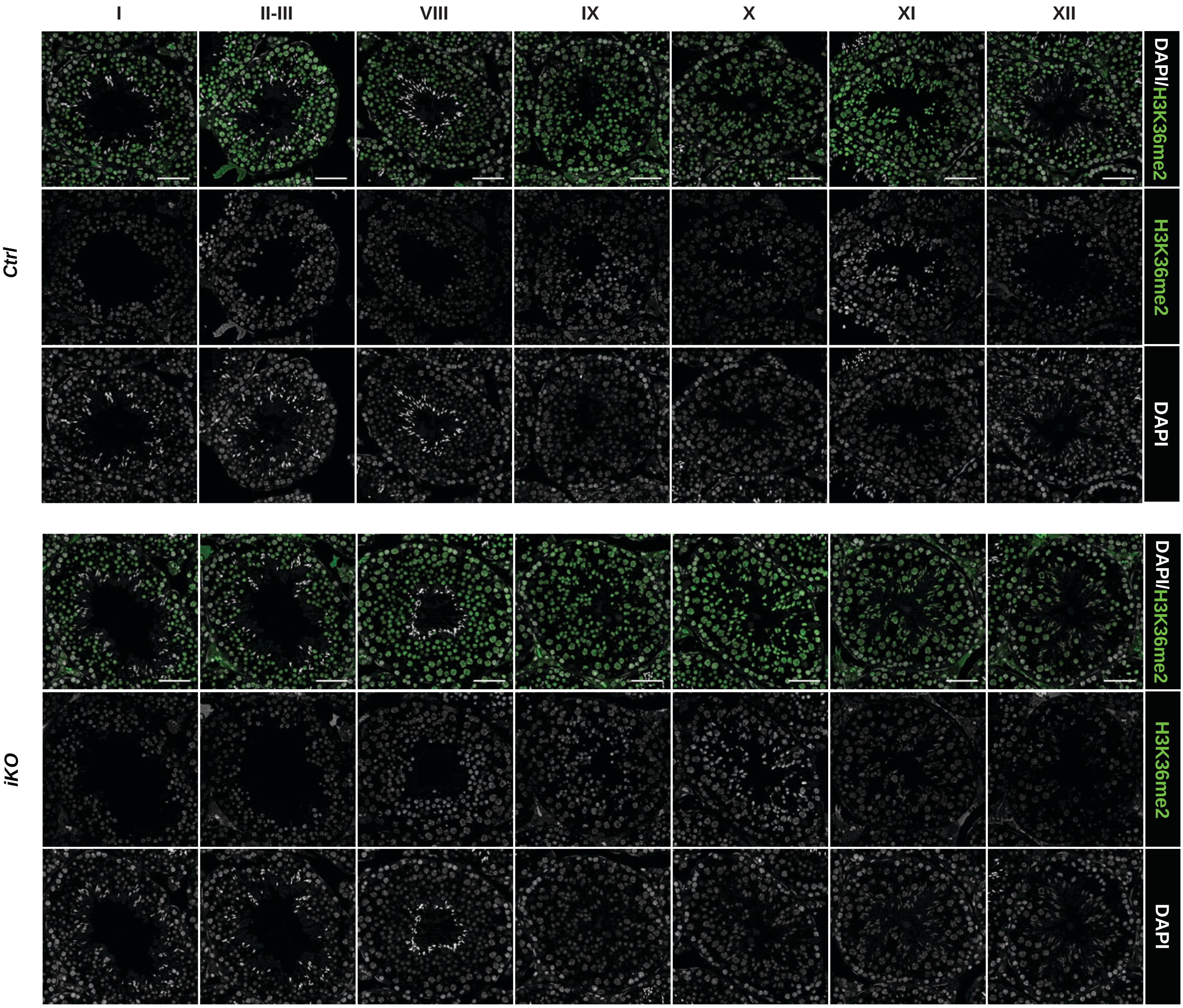

C

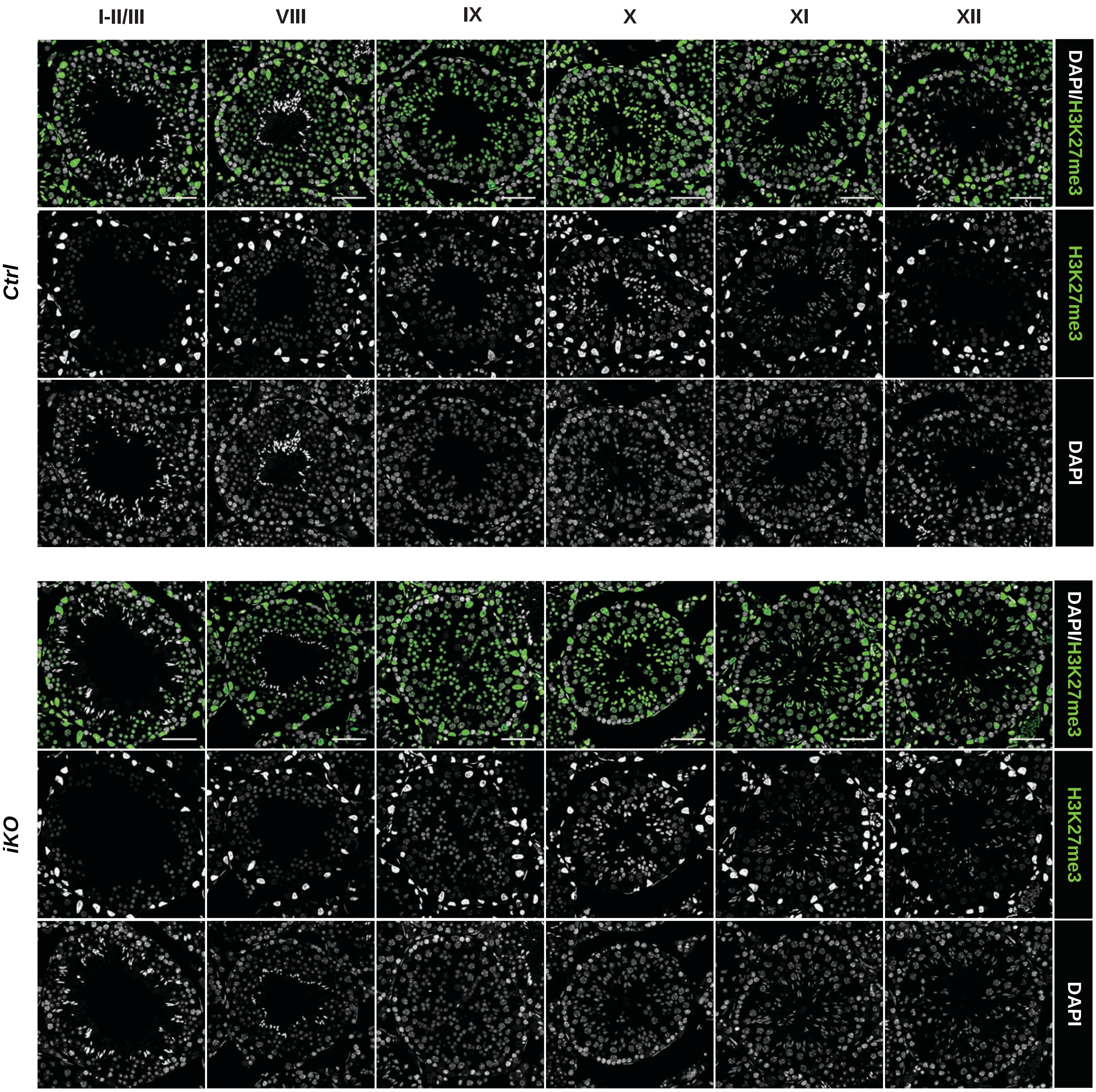

Supplementary Figure 14. Global levels of H3K36me3, H3K36me2 and H3K27me3 remain largely unaffected in *Kdm2a* iKO germ cells.

(A-C) Immunofluorescence staining of testis sections from Ctrl (*Kdm2a*$^{2lox/2lox}$) (n=1) and *Kdm2a* inducible knockout (iKO: *Kdm2a*$^{2lox/2lox}$; *CAGG-Cre-ER*^{TM}) (n=1) at 9d post first tamoxifen treatment, stained with (A) anti-H3K36me3 (B) anti-H3K36me2 and anti-H3K27me3 (green) antibodies. Nuclei were stained with DAPI (white). Representative images of various seminiferous tubule stages are shown. Scale bars = 50µm.
